# Towards Tricking a Pathogen’s Protease into Fighting Infection: The 3D Structure of a Stable Circularly Permuted Onconase Variant Cleavedby HIV-1 Protease

**DOI:** 10.1371/journal.pone.0054568

**Published:** 2013-01-18

**Authors:** Mariona Callís, Soraya Serrano, Antoni Benito, Douglas V. Laurents, Maria Vilanova, Marta Bruix, Marc Ribó

**Affiliations:** 1 Laboratori d’Enginyeria de Proteïnes, Departament de Biologia, Universitat de Girona, Girona, Spain; 2 Institut d’Investigació Biomèdica de Girona Dr. Josep Trueta, Girona, Spain; 3 Instituto de Química Física “Rocasolano”, Consejo Superior de Investigaciones Científicas, Madrid, Spain; National Research Council of Italy, Italy

## Abstract

Onconase® is a highly cytotoxic amphibian homolog of Ribonuclease A. Here, we describe the construction of circularly permuted Onconase® variants by connecting the N- and C-termini of this enzyme with amino acid residues that are recognized and cleaved by the human immunodeficiency virus protease. Uncleaved circularly permuted Onconase® variants are unusually stable, non-cytotoxic and can internalize in human T-lymphocyte Jurkat cells. The structure, stability and dynamics of an intact and a cleaved circularly permuted Onconase® variant were determined by Nuclear Magnetic Resonance spectroscopy and provide valuable insight into the changes in catalytic efficiency caused by the cleavage. The understanding of the structural environment and the dynamics of the activation process represents a first step toward the development of more effective drugs for the treatment of diseases related to pathogens expressing a specific protease. By taking advantage of the protease’s activity to initiate a cytotoxic cascade, this approach is thought to be less susceptible to known resistance mechanisms.

## Introduction

In Nature, zymogens and limited proteolysis are a common means to regulate the activity of proteases and of many other biological processes. This mechanism is based on the control of enzyme activation rather than inhibition [1]. Inspired by protease zymogens, Raines and co-workers extended this strategy to bovine pancreatic ribonuclease (RNase A) by connecting the amino and carboxyl termini with a peptide segment that caps the active site and thus acts like a pro-segment of a natural zymogen and inhibits the native ribonucleolytic activity [2], [3], [4]. Upon cleavage of this linker by a specific protease, near-wild type activity of RNase A is reconstituted. Despite their promise as targetable toxins, RNase A zymogens still must evade the action of the cytosolic ribonuclease inhibitor (RI), the natural regulator of ribonucleases in mammalian cells, that binds RNase A and its mammalian homologs with extremely high affinity (K_i_≈10^−15 ^M).

Onconase® (ONC), initially named P-30 protein [5] or ranpirnase, is an amphibian ribonuclease which has much less ribonuclease activity than its mammalian homologs but is much more cytotoxic [6], [7]. The high cytotoxicity of ONC has been attributed to a quick internalization process [8], [9], its ability to evade the cytosolic RI [7], [10], an unusually high conformational stability [11], [12] and to its ribonucleolytic activity [13], [14], [15]. Because of its cytotoxicity, ONC became the first ribonuclease to be assayed in clinical trials and is presently in advanced clinical trials for the treatment of different types of cancer [16], [17]. Further, ONC has been shown to inhibit human immunodeficiency virus 1 (HIV-1) production through the degradation of viral RNA [9]. Considering the special abilities of ONC, we reasoned that it would be interesting to construct zymogens of ONC with a linker sequence that obstructs the active site and encloses a cleavage site for HIV-1 protease. The first drugs available in the fight against Acquired Immune Deficiency Syndrome (AIDS) were nucleoside analogs designed to hamper reverse transcription; later peptide-based inhibitors aimed to block the action of HIV protease were developed (for reviews see [18], [19]. The combination of both types of drugs lead to the current highly effective antiretroviral therapy (HAART) which results in a dramatic reduction of virus burden with concomitant reduction in symptoms associated with the disease. Nevertheless, the development of drug resistance is an increasing problem and therefore new strategies that are less likely to be circumvented by the virus are needed.

Design and production of ONC-based zymogens would provide a mechanism to control the ribonucleolytic activity of ONC and consequently, of its cytotoxicity. Such an ONC zymogen could specifically target diseased cells that have the protease that activates the zymogen. Such a strategy could be further explored for the development of chemotherapeutics that could evade the known mechanisms of drug resistance developed by pathogenic organisms such as those observed for HIV-1 strains whose mutant proteases do not bind inhibitor compounds.

Here we report on ONC variants that can be cleaved by HIV-1 protease. Rational design has been used to construct and optimize circularly permuted variants of ONC that include the Gln1Ser substitution. Although treatment with the HIV-1 protease successfully cleaves some of the ONC variants, the linker lowers the catalytic efficiency of the ribonuclease, regardless of whether it is intact or proteolyzed, and a small increase in activity is observed upon proteolytic activation. To better understand the principles of the catalytic activity of our constructs, we have determined the solution structure of the best cleaved circularly permuted ONC variant, named ONCFLG, by NMR spectroscopy. The 3D structure of this circularly permuted ONC variant reveals insight into its activation and lays the foundation for further exploration of zymogens as promising tools for the treatment of HIV/AIDS or related diseases caused by retrovirus or other malignancies originated by pathogens harboring specific proteases.

## Materials and Methods

### Design of Circularly Permuted ONC Variants

To aid the design of circularly permuted ONC variants, structural models were created by modification of the atomic coordinates of wild type ONC (pdb accession code 1ONC) [20] and submitting a modeling request to the automated homology modeling server SWISS-MODEL. The models were optimized by using the GROMOS 96 implementation of Swiss-PdbViewer [21]. Only the retrieved models that were not distorted and did not showed global positive energy after minimization were considered for the development of circularly permuted ONC variants.

### Plasmid Construction

Plasmids that direct the production of ONC variants were constructed by circular permutation using pONC [22] as a template, following the strategy described by Raines and coworkers [2]. The detailed procedure and the oligonucleotides are available in the **Supporting Information S1 file**.

### Production of Circularly Permuted ONC Variants, ONCQ1S, Wild Type ONC and HIV-1 Protease

Circularly permuted ONC variants and ONCQ1S were produced and purified essentially as described previously for wild type ONC [8], [23]. Production and kinetic characterization of HIV-1 protease has been described in detail in the **Supporting Information S1** file.

### Calorimetric Conformational Stability Determination

The stability of wild type ONC, ONCQ1S, ONCFL and ONCFLG was determined by differential scanning calorimetry (DSC) at the Plataforma de Polimorfisme i Calorimetria from the Serveis Cientificotècnics of the Universitat de Barcelona by means of a VP-DSC equipment from MicroCal (Northampton, MA, USA). For these measurements, proteins were dissolved in 0.1 M 2-[N-morpholine] ethanesulfonic acid-NaOH (MES-NaOH) buffer, pH 6.0 at concentrations of 0.15–0.18 mM as determined by Bradford assay [24], and temperature was increased from 30 to 110°C using a scan rate of 1 K min^−1^. *T*_d_ is the denaturation temperature and corresponds to the maximum of the DSC peak.

### Activation of Circularly Permuted ONC Variants

The activation of the different circularly permuted ONC variants was assayed with different molar ratios of the HIV-1 protease to variant ranging from 1∶5 to 1∶100, in buffer composed of 100 mM sodium acetate/acetic acid, pH 4.7, 300 mM NaCl and 4 mM EDTA. Aliquots were taken at different times, mixed with SDS-PAGE loading buffer and stored at −20° until analysis by electrophoresis. Progress of the activation reactions was quantified using the Imaging System FluorChem® SP (Alpha Innotech, San Leandro, CA, USA).

### Enzymatic Activity of Circularly Permuted ONC Variants

The ribonucleolytic activity of ONCFL and ONCFLG variants before and after cleavage was measured with the fluorogenic substrate 6-FAM-dArUdAdA-6-TAMRA [25] using a Lambda LS50 fluorescence spectrometer (Perkin-Elmer, Waltham, MA, USA) equipped with a thermostat and sample stirring. Each measurement was repeated at least three times and values in **Table 1** are expressed as the mean ±SD.

**Table 1:** Analysis of the stability and functionality of ONC variants.

	T_d_(°C)^a^	IC_50_ (µM)^b^ uncleaved	*k*_cat_/K_M__uncleaved_ c	*k*_cat_/K_M__cleaved_ c	*k*_cat_/K_M__cleaved_/*k*_cat_/K_M__uncleaved_
wild type ONC	87.5±0.3	0.46±0.03	–	7.31±0.11	–
ONCQ1S	84.7±0.5	4.0±0.8	–	2.60±0.08	–
ONCFL	80.9±0.1	28.2±0.9	0.37±0.04	0.47±0.06	1.27
ONCFLG	80.5±0.1	8±2	0.95±0.16	1.10±0.04	1.16

a T_d_ were determined in 0.1 M MES-NaOH buffer, pH 6.0 by DSC as described in Material and Methods.

b IC50 (±SD) values were determined using the CellTiter96® cell viability assay as described in Material and Methods.

c Units of catalytic efficiency, *k*_cat_/K_M_, are 10^2^ M^−1^s^−1^. Values of *k*_cat_/K_M_ were determined for catalysis of 6-FAM-dArUdAdA-6-TAMRA cleavage at 25°C in 0.1 M MES-NaOH buffer, pH 6.0, containing 0.1 M NaCl [43].

### Cytotoxicity of Circularly Permuted ONC Variants

The cytotoxicity of wild type ONC, ONCQ1S and circularly permuted ONCFL and ONCFLG variants was assayed on human T-lymphocytes Jurkat cells by measuring the IC50 values as described previously [8]. The IC_50_ values represent the concentration of the assayed enzyme required to inhibit cell proliferation by 50%. The results for a single experiment are the average of three determinations, and the experiments were repeated independently three times each (**Table 1**).

### Internalization of ONCFLG-Cys Variant

The Ser72Cys substitution was introduced by QuikChange™ (Stratagene, La Jolla, CA) site-directed mutagenesis at the C-terminus of ONCFLG variant. This Cys was conjugated to the fluorochrome Alexa Fluor® 488 C5 maleimide (Molecular Probes, Life Technologies, Carlsbad, CA, USA) essentially as recommended by the manufacturer. Jurkat cells were incubated with the labeled variant for known times, and in all samples, cell nuclei and membranes were counterstained with Hoechst 33342 (Molecular Probes, Eugene, OR, USA) and DiI, respectively. Considering that Jurkat cells are an immortalized line of T lymphocytes, a common host of HIV infection, they are relevant for studies aiming to develop improved AIDS therapeutics. Internalization was visualized with a Leica TCS SP2 AOBS laser scanning confocal microscope at the Servei de Microscòpia de la Universitat Autònoma de Barcelona.

### Solution Structure, Dynamics and Conformational Stability Determined by NMR

The solution structure of ONCFLG (pdb: 2LT5) at pH 5.2 35°C was determined on the basis of NOE distance constraints and backbone torsional angle restrictions. ^1^H-^2^H exchange to monitored conformational stability was monitored by a series of ^1^H-^15^N HSQC experiments. The ^1^H-^15^N internuclear NOE was measured to gauge the residue-level dynamics. The complete procedural details of all these experiments as well as the interaction of ONCFLG with the d(UGG)_3_ substrate analog and the determination of a structural model of the cleaved form of ONCFLG are given in the **Supporting Information S1** file.

### Accession Numbers

The ^1^H, ^13^C and ^15^N resonance chemical shift assignments of ONCFLG variant have been deposited at the BMRB with the deposition number 17973 and the coordinates are available at the Protein Data Bank with the accession code 2LT5.

## Results

### Design, Production and Activation of ONCYP Variant

When designing ONCYP, two features of ONC that might influence the activity, namely, the overall stability and the cytotoxicity, were taken into account. First, the N-terminal Gln1 of ONC, which spontaneously cyclizes to form a pyroglutamyl (Pyr) residue [13], [20], was replaced by Ser, which is the substitution that retained the highest catalytic efficiency and cytotoxicity among those surveyed [14]. Second, ONC presents a C87/C104 disulfide bond that tethers the C- terminal Cys104 to a central β-strand, and thus sequesters it from the N-terminus [20]. Analysis of the crystal structure of ONC (pdb code 1ONC) reveals that the distance between the N- and C-terminal residues is 18 Á. This distance could be covered by as few as 5 residues in a fully extended conformation. Our simulations, however, suggested that sequences of 14 amino acids or longer would be required to connect the native termini and encode the HIV protease recognition motif without perturbing the overall structure.

**Figure 1:**
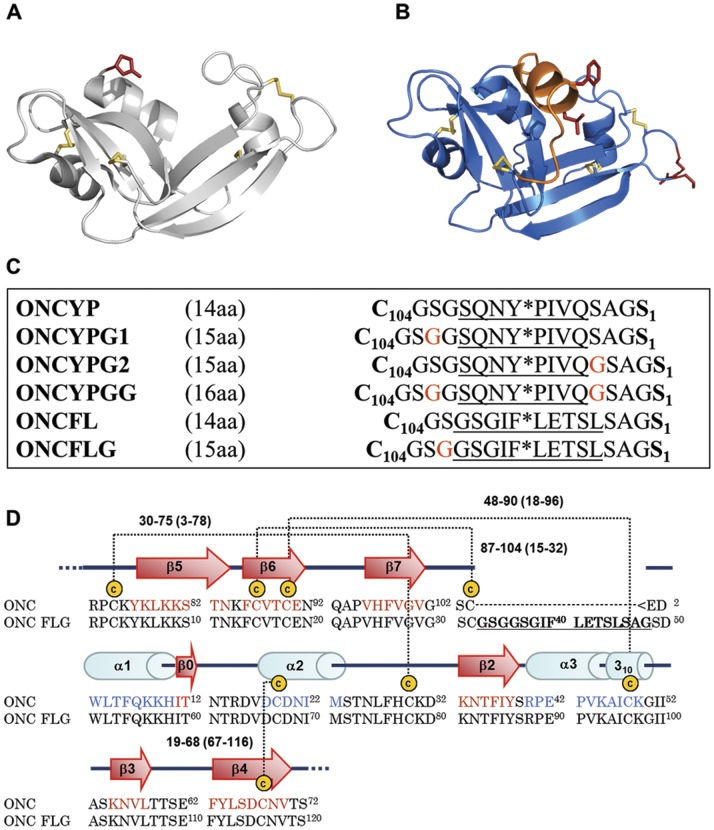
Zymogen model and linker sequences. (A) 3D structure of ONC (pdb accession code, 1ONC). (B) Structural model of the ONCFL variant generated using the automated homology modeling server SWISS-MODEL and energy minimized with the GROMOS 96 implementation of Swiss-PdbViewer [21]. The amino and carboxyl termini created by circular permutation and the residues that constitute the cleavage site are represented using red sticks. Both figures were drawn with PyMOL (http://pymol.sourceforge.net/). Note that the linker segment was predicted to be helical in this model, but its structural characterization by NMR shows that it is disordered. (C) Scheme of the primary sequence of the linkers used in the design and construction of the different zymogens. Native carboxyl and amino residues of ONC are in bold and Gly residues used to elongate the linker are in red. The HIV-1 protease recognition sequence is underlined and Y*P and F*L indicate the hydrolyzed peptide bonds. (D) Alignment of ONC and ONCFLG amino acid sequences showing the secondary structure representation for ONC. The residues that compose the FLG linker connecting Ser1 and Cys104 are in bold and underlined.

The selection of the new termini Arg73/Ser72 was based on the solvent accessibility of these residues, which form a loop connecting strands β4 and β5 of ONC. We had previously shown that this loop is able to accommodate a Ser72Cys substitution, which we used to fluorescently label ONC and follow its cellular internalization [8]. Moreover, in many onconase homologs, e.g. Eosinophil Cationic Protein [26] this loop is much longer. This fact, and the observation of flexibility in this loop as judged by normal mode analysis and conformational changes upon ligand binding or temperature changes [15] suggests that this loop will be tolerant to modification. In contrast, two other solvent exposed loops, that connect the last two β-strands or the third and second to last β–strands in wild type onconase, frequently contain cis Prolines in onconase homologs such as RNase A and are much less tolerant to mutation. Finally, thanks to the disulfide bonds 19–68 and 30–75 in wild type onconase, the new free N- and C-termini will still be effectively tethered to the rest of the protein in circularly permuted variants despite the rupture of the protein chain between residues 72 and 73. The X-ray structure of wild type ONC and a model of ONCFLG (generated as described in Material and Methods section) highlighting the old and new termini are shown in **Figure 1** together with the lengths and the sequences of the different linkers used in this work and the sequence numbering of ONC and the ONCFLG variant.

In the ONCYP variant, the native termini, Cys104 and Ser1, are connected by the octapeptide sequence SQNY*PIVQ, which corresponds to the naturally occurring Pr55gag p17/p24 cleavage site for HIV-1 protease where Tyr*Pro is the cleaved peptide bond [27]. This sequence is also present in the substrate used to verify the protease’s activity following its purification (see **Supporting Information S1**). The residues flanking the recognition sequence of HIV-1 protease (**Figure 1**) were intended to link the protein ends while providing flexibility and solubility due to their small and/or polar side-chains. After expression in *E. coli* BL21 (DE3) as inclusion bodies, the ONCYP variant was subjected to oxidative refolding *in vitro* and purified with a yield exceeding 25 mg/L of culture. Protein purity and homogeneity were confirmed by SDS-PAGE and the molecular mass confirmed by MALDI-TOF mass spectrometry (**Table S1**).

**Figure 2:**
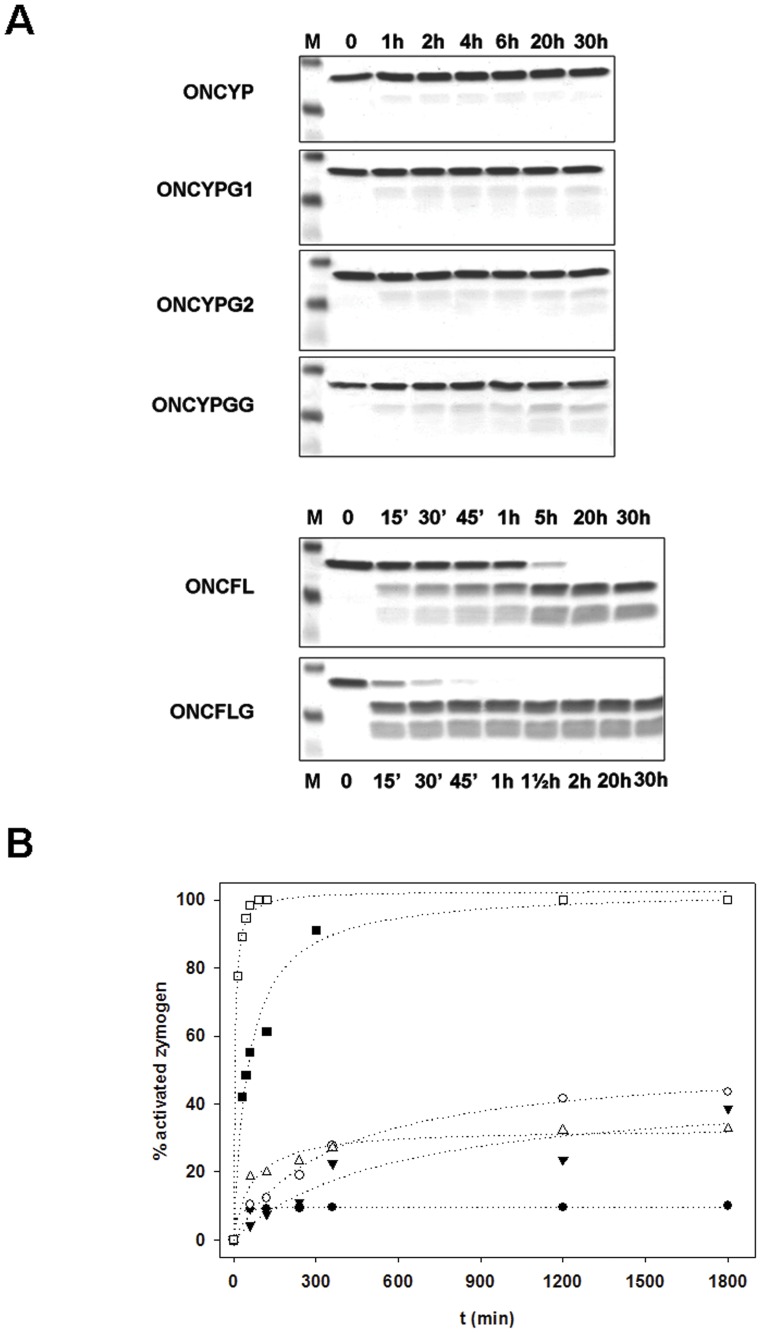
Cleavage of circularly permuted ONC variants by HIV-1 protease. (A) Cleavage measured at different times after addition of HIV-1 protease at a molar ratio (protease:variant) of 1∶5 for YP and 1∶100 for FL variants and analysis by Coomasie-stained SDS-PAGE under reducing conditions. For all the gels, lane M corresponds to molecular mass standards: 15, 10 and 4 kDa. For variants ONCYP, ONCYPG1, ONCYPG2 and ONCYPGG, lanes 1 to 7 correspond to samples taken 0, 1, 2, 4, 6, 20 and 30 h after protease addition. For ONCFL (lanes 1–8), samples were taken at 0 min, 15 min, 30 min, 45 min, 1 h, 5 h, 20 h and 30 h and for ONCFLG (lanes 1–8), at 0 min, 15 min, 30 min, 45 min, 1 h, 1.30 h, 2 h, 20 h and 30 h. (B) Time-course of the activation of the different variants followed by quantification of the disappearance of the variant band using the Imaging System FluorChem® SP (Alpha Innotech, San Leandro, CA, USA). Legends: ONCYP (•); ONCYPG1 (▾); ONCYPG2 (δ); ONCYPGG (○); ONCFL (▪); ONCFLG (□).

The cleavage of the ONCYP by HIV-protease, was assayed using different molar protease:ONC variant ratios and distinct incubation times. As shown in **Figure 2**, the cleavage of ONCYP rapidly reached a plateau approximately 4 hours after the addition of protease. Even when a 1∶5 molar protease:ONC variant ratio was used, further incubation (up to 30 hours) did not result in additional cleavage, and the final yield of cleaved variant was only 10%.

### Circularly Permuted Variant Cleavage Optimization

With the aim of improving protease action, new linkers (**Figure 1**) were designed based on two principles: i) flexibility and linker length were increased to favor the accessibility of the protease and ii) a different protease recognition motif sequence was employed. An additional Gly residue was added either on one side (ONCYPG1) or the other (ONCYPG2) or on both sides (ONCYPGG) of the cleaved bond (**Figure 1**). Secondly, we choose GSGIF*LETSL as a new target sequence [28]. Because the 14 residue linker of ONCYP resulted in poor cleavage, ONCFL and ONCFLG variants were designed with linkers of 15 and 16 residues, respectively. As shown in **Figure 1**, ONCFLG contains an additional Gly residue on the amino side of the bond to be cleaved.

The activity of HIV-1 protease on these new ONC variants was also assayed. As shown in **Figure 2**, after 30 hours of incubation with a 1∶5 protease:ONC variant ratio, 38%, 32% and 43% of ONCYPG1, ONCYPG2 and ONCYPGG, respectively, were cleaved. Thus, elongation of the linker sequence results in 3–4 fold increase in cleavage relative to the original ONCYP variant containing the matrix/capsid (MA/CA) cleavage site of the HIV-1 Gag-Pol polyproteins. More interestingly, ONCFL and ONCFLG variants were completely cleaved at shorter incubation times (**Figure 2A**) and using molar ratios of HIV-1 protease:ONC variant as low as 1∶100. These results indicate that linkers based on the amino acid sequence GSGIF*LETSL are more efficiently hydrolyzed by HIV-1 protease than those based on the MA/CA site SQNY*PIVQ.

### Catalytic Efficiency of the Intact and Cleaved Circularly Permuted Variants

Next, the functionality of ONCFL and ONCFLG variants was determined by measuring the ribonucleolytic activity on the fluorogenic substrate 6-FAM-dArUdAdA-6-TAMRA of both the precursor and the cleaved form of each variant. As shown in **Table 1**, treatment with HIV-1 protease marginally increased the ability of both variants to catalyze the cleavage of the tetranucleotide substrate by approximately 1.27 and 1.16-fold for ONCFL and ONCFLG, respectively. Therefore, the low catalytic efficiency observed for the intact and cleaved variants relative to the Q1S variant is not the result of the intact linker, but rather the additional amino acids at the termini, regardless of whether they are connected or not.

### Cytotoxicity of Circularly Permuted ONC Variants

The cytotoxicity of uncleaved ONCFL and ONCFLG variants was compared to wild type ONC and ONCQ1S by measuring their IC_50_ values on human T-lymphocytes Jurkat cells. Both variants were less toxic to Jurkat cells than the parent enzymes and their IC_50_ values increased between 2- and 70-fold in comparison to the IC_50_ values of ONCQ1S and ONC, respectively (**Table 1** **)**. These results indicate that these variants lose some of the cytotoxicity of the wild type enzyme.

### Internalization of ONCFLG-Cys Variant

ONC internalization into K562 erythroleukemic cells has been shown to proceed rapidly and maximum cell labeling was achieved after only 45 min [29]. To evaluate the internalization capability of circularly permuted ONC variants, ONCFLG variant in which the C-terminal Ser was replaced by Cys, was fluorescently labeled by conjugation with Alexa Fluor® 488 C5 maleimide. Alexa-labeled ONCFLG variant was incubated with Jurkat cells and internalization was monitored at different times in samples with counterstained cell nuclei and membranes. As shown in **Figure S1**, labeled variant is detected inside the cells after 3–6 h and internalization increased up to 12 h after incubation using concentration as low as 2 µM of labeled variant.

### Structure of ONCFLG Variant

**Figure 3:**
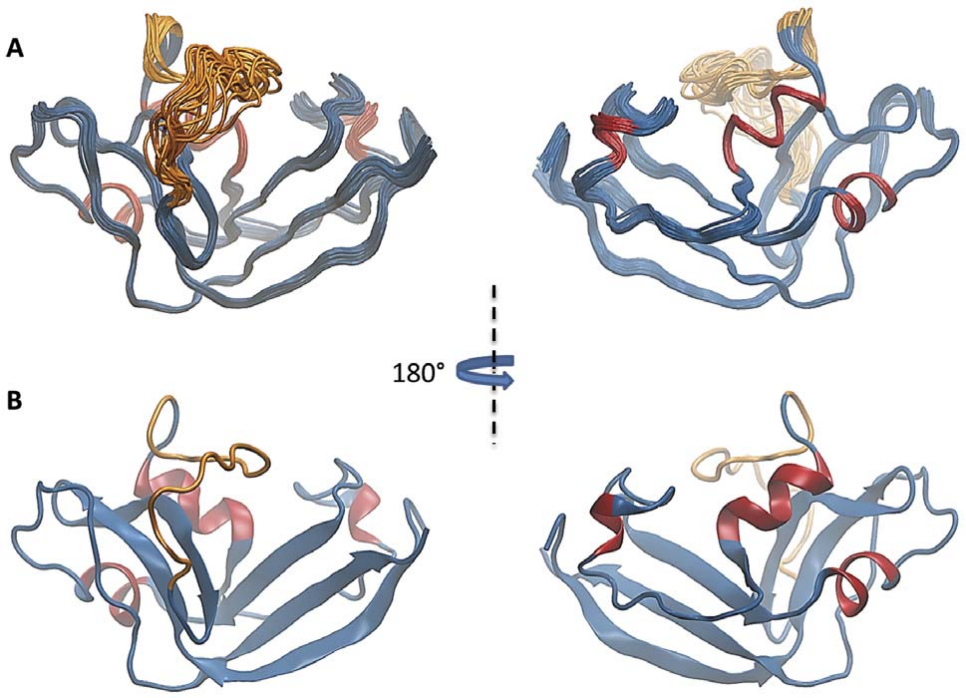
3D structure of ONCFLG by NMR. (A) Backbone superposition of the 20 lowest energy conformers of ONCFLG. The disordered linker segment (Gly33-Gly48) is represented in orange and the rest of the protein backbone sequence in blue except for the α-helices which are in red. The views are rotated 180° with respect to each other. (B) Ribbon drawing of a representative conformer of the energy-minimized family of ONCFLG variant structures. Colors and the orientation are the same as in (A). This figure was generated by VMD [41].

The superposition of the 20 conformers of the best cleaved variant, ONCFLG, determined by NMR is shown in **Figure 3**. These structures, obtained utilizing the calculation protocol described in the Material and Methods section, satisfy the structural constraints and have low potential energies (**Table 2**). The structure is well defined, maintains the same tertiary structure shown by other members of the RNase A superfamily and is particularly similar to that of wild type ONC [20], [30], and also to variants prepared by site-directed mutagenesis [31], [32] and cytotoxic bullfrog ribonucleases [33], [34]. The three α-helices: α1, Leu52-His58, α2, Ile70-Ser72 and α3, Pro89-Ala94 (variant numbering) match quite well with the helices present in wild type ONC consisting of residues Trp3-His10, Cys19-Ile22, Pro41-Lys49 (wild type numbering). The β-strands β5, Tyr5-Asn12, β6, Phe14-Cys18, β7, Pro23-Val29, β0, Ile59-Thr60, β2, Lys81-Tyr86, β3, Lys103-Thr107 and β4, Phe111-Val118 are also equivalent to those of native ONC. A detailed inspection of ONCFLG structure shows that there is a subtle local rearrangement near Cys18-Cys96 respect to wild type ONC. In ONCFLG, there is an increase of about 1Å between these cysteines’ Cα -Cα distance that is probably related with the absence of the 3_10_-helix motif following α3 present in wild type ONC (**Figure S2**).

**Table 2:** Structural Statistics of the 20 best NMR Structures of the ONCFLG.

NOE Distance and Dihedral Constraints		
N° short-range distances (|i–j|≤1)		1059
N° medium-range distances (1<|i–j|<5)		288
N° long-range distance (|i–j|≥5)		678
N° restraints from S-S bonds		24
N° angular restraints (φ,ψ)		149
N° total restraints		2198
N° restrictions/residue		18.3
**Structure Calculation**		
CYANA target function value (min, mean, max)		1.54, 1.69, 1.96
Maximum distance violation (Å) (min, mean, max)		0.12, 0.20, 0.47
Maximum dihedral angle violation (°) (min, mean, max)		2.32, 3.58, 9.51
Average AMBER energy (kcal/mol)		−3926
Average AMBER Electrostatic energy (kcal/mol)		−7769
RMSD bond lengths from ideal geometry (Å)		0.01±0.001
RMSD angles from ideal geometry (°)		2.17±0.01
**Averaged pairwaise RMSD (Å) (backbone, heavy atoms)**		
Global (1–120)	0.8±0.3	1.3±0.3
Structured region (1–32, 49–120)	0.32±0.07	0.96±0.07
Unstructured linker (33–48)	1.5±0.6	2.1±0.8
Secondary structure (helices and sheets)	0.25±0.06	0.84±0.08
α-helices (51–58, 70–72, 90–94)	0.21±0.06	1.0±0.2
β-sheets (5–12, 14–18, 23–29, 59–60, 81–86, 103–107, 111–118)	0.23±0.05	0.75±0.09
**Ramachandran Plot Analysis (%)**		
Most favored regions	74.9	
Additional allowed regions		24.7
Generously allowed regions		0.0
Disallowed regions		0.0

It is interesting that the linker segment Gly33-Gly48 designed to block the access to the active site of the ONCFLG variant does not adopt a regular secondary structure. The backbone RMSD of this segment is 1.5 Å in contrast to 0.2 Å shown by the rest of the protein (**Table 2**). This region extends beyond of the protein core occupying the concave face of the common RNase kidney-shaped structure. In this way, access to the active site cleft containing the catalytic triad (His25, His58 and Lys79) that is characteristic of the RNase A superfamily and also, Ser49 (Pyr1 in wild type) and Lys57, exclusive of frog ribonucleases, may be hindered by this flexible linker, which may act as a “steric bristle” [35].

**Figure 4:**
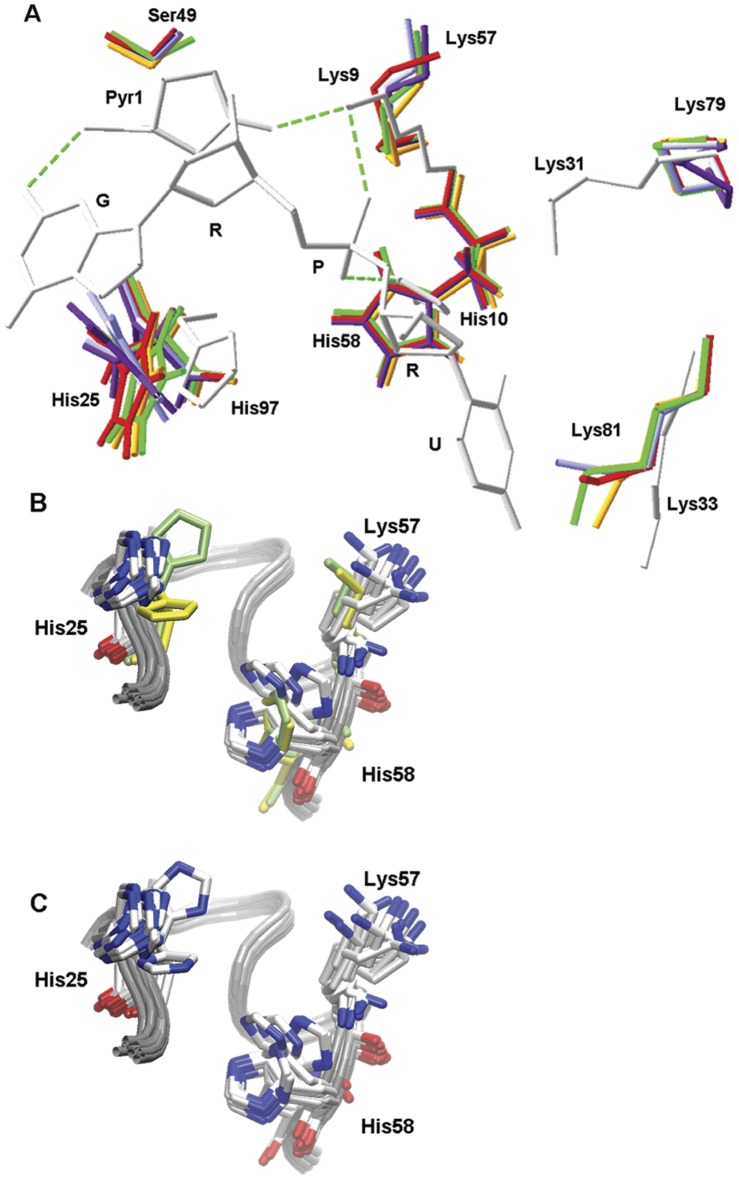
Superposition of different conformations of the active site residues. A. Comparison of different conformations of ONCFLG P1 subsite residues shown in different colors with the homologous residues from ONC-d(AUGA) in white. Letters G, R, P and U stand for guanine, ribose, phosphate and uracil, respectively. B. ONCFLG conformations are shown in CPK color while wild type ONC (1ONC.pdb) and ONC-d(AUGA) complex (2I5S.pdb) are shown in lime and in yellow, respectively. C. Same as B except that all the residues are drawn in CPK color to show how Nδ1 and Nε2 from His25 exchange their position through the rotation around the χ2 dihedral angle, midway between those of His97A conformation observed in the ONC-d(AUGA) complex and the His97B conformation shown in wild type ONC structure. Structural alignments and figures B and C and were generated using VMD (Humphrey, Dalke et al. 1996), whereas figure A was drawn using Swiss-PdbViewer (Guex and Peitsch 1997).

A closer look at the structure provides information about the active site residues and their relationship to the ribonucleolytic activity. Whereas all the residues belonging to B1 (Lys81, Thr83, Asp115 and Phe26) and B2 (Thr17 and Glu19) subsites show the same orientation as the equivalent residues in the crystal structure of wild type ONC (1ONC) [20] or ONC-d(AUGA) complex (2I5S) [31], remarkable differences are observed for active site (or P1 subsite) residues. Ser49 Oγ, that should replace the wild type N-terminal Pyr1 Oε, is not hydrogen-bonded to Lys57 Nξ (Lys9 in wild type ONC) and consequently a high mobility is observed for Lys57 side chain. Lys79 has been proposed to work together with Lys57 to stabilize negative charge buildup on a nonbridging phosphoryl oxygen during catalysis [13], [20], [31]. However, Lys79 protrudes out of the ONCFLG structure, instead of pointing towards the active site cleft as does its equivalent Lys31 in wild type ONC (**Figure 4**). As a consequence of the movement of Lys57, His58 appears to oscillate up and down in the ONCFLG structure between two different conformations which differ by approximately 90° in the occupancy of the imidazole ring. The imidazole ring of His25, in contrast, occupies an ensemble of conformations midway between those of the His97A conformation observed in the ONC-d(AUGA) complex (2I5S) and the His97B conformation shown in wild type ONC (1ONC) structure (**Figure 4B and 4C**).

### Interaction of ONCFLG with a Substrate Analog

To further characterize the active site of ONCFLG, we studied its interaction with an uncleavable substrate analog, d(UGG)_3_. The complete details of these experiments are provided in the **Supporting Information S1 file**. In summary, ONCFLG and d(UGG)_3_ combine immediately at pH<7 to form an insoluble precipitate, but remain dissolved about pH 7 with little evidence for specific binding. The formation of the precipitate at low pH is likely to be driven by relatively nonspecific electrostatic interactions. At alkaline pH, no unambiguous intermolecular NOEs between d(UGG)_3_ and ONCFLG could be assigned, no changes in the tautomeric states of the His residues were observed and only two significant chemical shift changes were detected. Therefore, at alkaline pH the binding of ONCFLG and d(UGG)_3_ appears to be weak to insignificant.

### Structure of the Cleaved ONCFLG Variant

**Figure 5:**
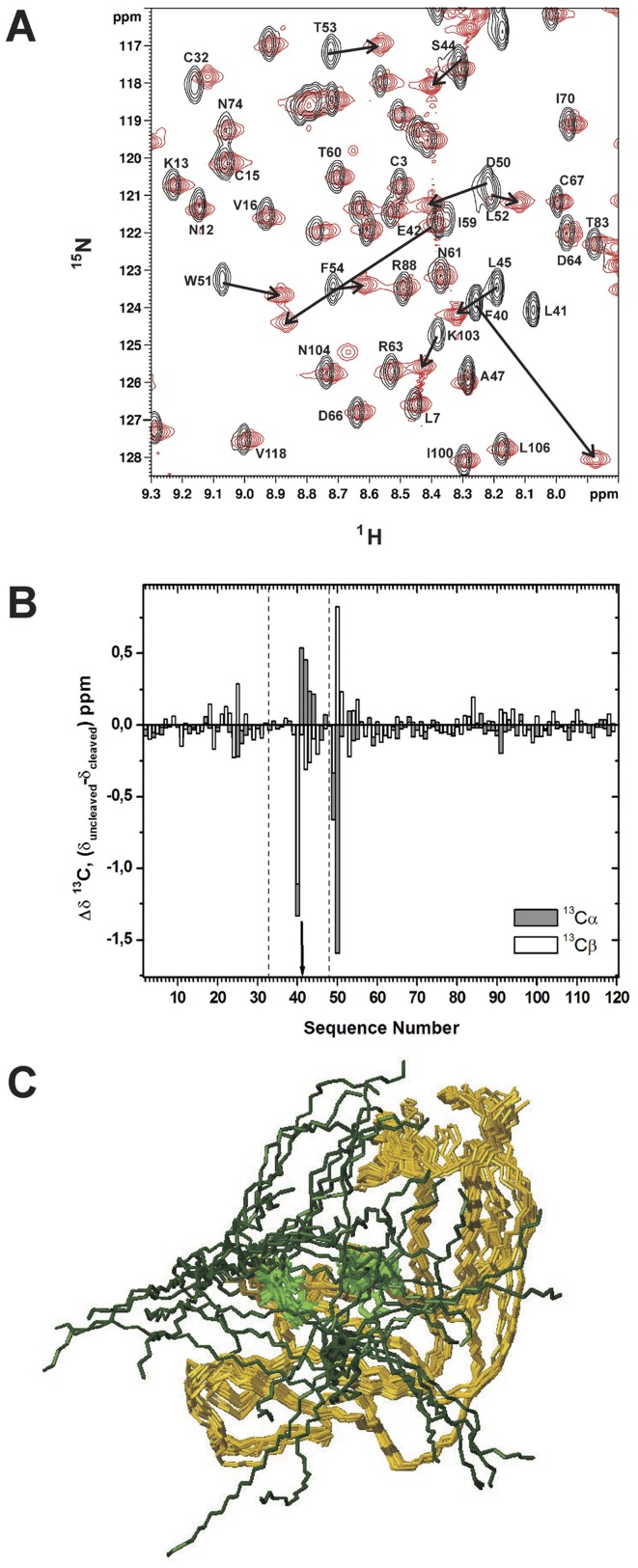
NMR data and structural model of the cleaved ONCFLG variant. (A) Region of the 2D ^1^H-^15^N HSQC spectra of the ^15^N labeled ONCFLG variant (black) superimposed onto that of cleaved form (red). Spectra were recorded at 800 MHz, 35°C and pH 5.2. Representative ^1^H-^15^N correlations of the uncleaved form are labeled and black arrows indicate the corresponding shifted signals in the cleaved ONCFLG form. (B) Comparison of the ^13^Cαand ^13^Cβ chemical shift values for the uncleaved and cleaved forms of ONCFLG variant. Δδ values were obtained from the difference between the reported uncleaved (BMRB n°: 17973) and cleaved ONCFLG forms values (Δδ = δ _uncleaved_ – δ _cleaved_). The arrow shows the position of the hydrolyzed peptide bond, and the linker segment, Gly33-Gly48, within the dashed lines. (C) Structural model of the cleaved ONCFLG form. The cleaved linker segment (Gly33-Gly48) is represented in dark green and the rest of the protein backbone sequence in dark yellow. The active site histidines are in light green. This figure was generated by MOLMOL [42].

To determine the structural changes in ONCFLG provoked by HIV-1 cleavage, the resonance assignments of the cleaved variant were obtained following the same methodologies used for the uncleaved protein [36]. **Figure 5A** shows a comparison of the ^1^H-^15^NHSQC of the cleaved and uncleaved forms of ONCFLG. As expected, the HN signal corresponding to Leu41 is absent in the cleaved form, as hydrolysis by the HIV-1 protease of the Phe40-Leu41 amide bond converts the CO-HN group into –COO^–^ and H_3_N^+^–. Most of the HN signals retain their chemical shifts upon cleavage, and the most affected ones belong to Phe40 and Glu42, which flank the HIV-1 protease cleavage site. The differences of the ^13^Cα and ^13^Cβ chemical shifts (Δδ) between the cleaved and uncleaved ONCFLG variant are plotted in **Figure 5B**. Most Δδ values are small (<0.1 ppm) except for the segment Phe40-Asp50 located close to the hydrolyzed peptide bond. These data clearly point to the conservation of the global fold, accompanied by structural differences in the linker segment after cleavage. Moreover, the analysis of the NOESY spectra allowed us to identify similar sets of short and long range NOEs in the structured regions of the cleaved and uncleaved variant. This corroborates that their 3D structures are very similar. On the basis of these data, a structural model for the cleaved ONCFLG variant was built (**Figure 5C**). The most remarkable change is the increased number of conformations available to the segments bordering the cleavage site.

### ONCFLG Variant Dynamics

**Figure 6:**
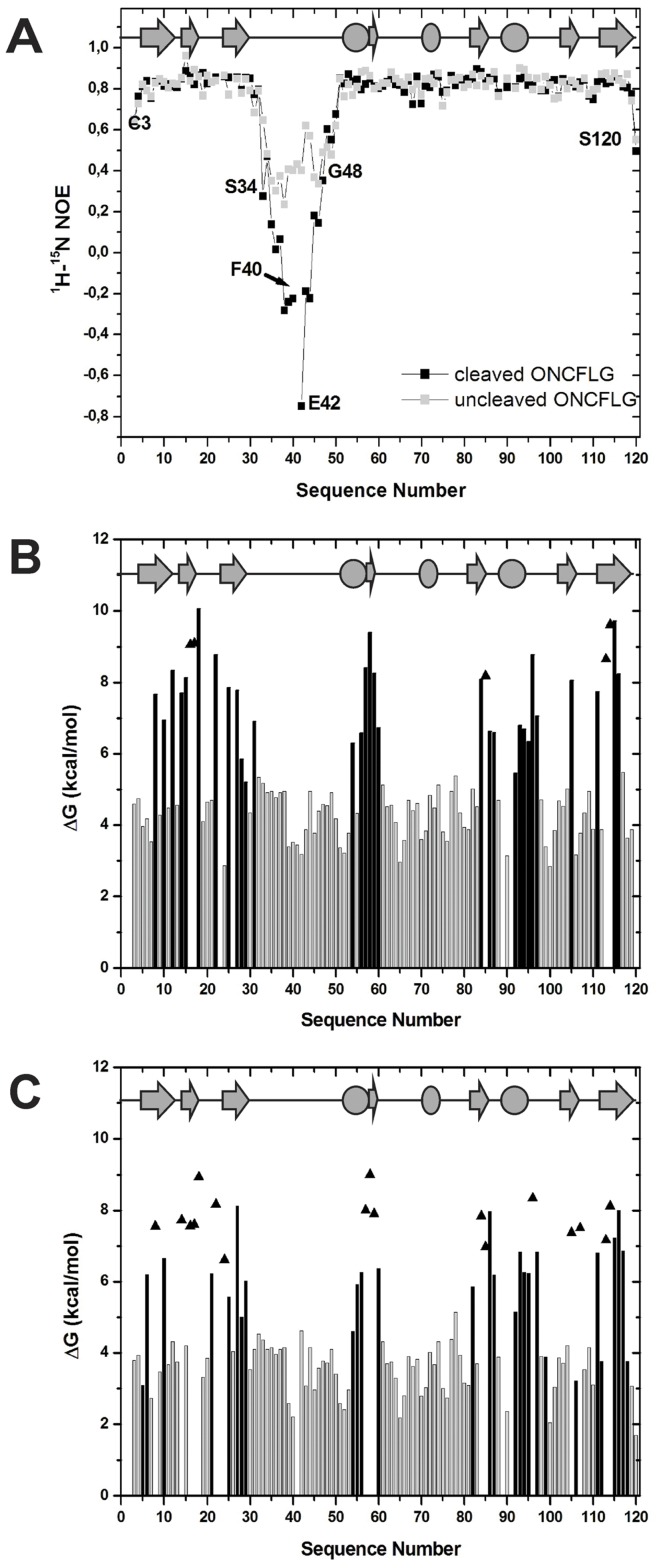
Dynamics and conformational stability of the ONCFLG variant forms. (A) The ^1^H-^15^N NOE ratio for the uncleaved (gray) and cleaved ONCFLG forms (black) as a function of the sequence number. Lines connecting gray or black squares boxes are used to guide the eye. (B) and (C) ΔG_HX_ values obtained by NMR-monitored ^1^H-^2^H exchange of the uncleaved (B) and cleaved (C) forms of the ONCFLG as a function of the sequence number. Grey bars represent the upper limits for fast exchanging groups, black bars show experimentally measured values and triangles indicate the estimated lower limit for slow exchanging groups. The secondary structure is represented on the top of each panel, β-strands as gray arrows and α-helices as ovals.

To elucidate the local backbone flexibility of ONCFLG variant in the ns-ps time-scale, we have measured the heteronuclear NOEs (**Figure 6A**). Most residues show NOE ratio values close to the highest value (0.85) expected theoretically for rigid ^15^N-^1^H vectors, in agreement with previously reported results in ONC and its variants [33], [37]. The N- and C-termini are more flexible with NOE ratio values ranging from about 0.5 to 0.6. The heteronuclear NOE values show that the 16 residue segment inserted to connect the wild type termini is the most flexible part of the molecule. In the uncleaved form, residues Ser34 to Gly48 show an average NOE ratio value of 0.38. This suggests that this region behaves as a flexible loop with fast motions in the ns-ps time scale characteristic of an unstructured region. Remarkably, this flexibility is substantially increased in the cleaved form with Gly38 to Ser44 showing negative NOE values. Thus, the conformational freedom of the new termini is like that of short unstructured peptides.

### Conformational Stability of the Circularly Permuted ONC Variants

As shown in **Table 1**, the midpoint of thermal unfolding (T_d_) of the ONCFL (80.9°C) and ONCFLG (80.5°C) variants as determined by DSC, are considerably higher than the physiological temperature (37°C) and comparable to the values determined previously for ONCQ1S and wild type ONC [38]. This ensures that these variants are stable enough to be used *in vitro* and *in vivo* under physiological conditions.

Moreover, the residue-level conformational stability of ONCFLG in the uncleaved and cleaved forms, ΔG_HX_, was measured by ^1^H-^2^H exchange monitored by NMR. The most protected and stable amide groups (**Figure 6B**) are located in the secondary structure regions, mainly the β-sheets and the first and third α-helices. Relative to most proteins, including RNase A [39], the average ΔG_HX_ of these secondary structural elements is high, which reflects the extraordinary conformational stability of ONCFLG. Remarkably, the inserted segment (residues 33–48) shows no measureable protection against ^1^H-^2^H exchange under these conditions. The HN protons of the second helix are also unprotected as was described for wild type ONC and variants [37]. The same helix has been observed to have a relatively low stability in RNase A and human RNase 3 (eosinophil cationic protein) [26]. The geometry of the H-bonds formed by this helix is suboptimal in these proteins. Upon cleavage, the ΔG_HX_ values become about 1.7 kcal/mol lower on average. Assuming that the dependence of the free energy of stability on T_d_ observed for wild type ONC [29] holds for ONCFLG, we calculate that the cleavage of ONCFLG would lower its T_d_ about 6.6°C to 73.9°C. This T_d_ is still far above 37°C, so cleaved ONCFLG will be stably folded at physiological temperatures (**Figure 6C**).

## Discussion

To create a ribonuclease zymogen [2] the bridge connecting the N- and C-termini of the native enzyme must extend over and block access to the active site. The zymogen is activated when a specific protease recognizes and cleaves a specific motif within the bridge. Therefore, the bridge segment must be long enough to connect the termini without inducing strain and to encode the protease recognition motif, but short enough to effectively fill the active site groove to prevent the RNA substrate from binding the uncleaved zymogen.

The initial designed circularly permuted ONC variant, ONCYP, was not properly cleaved by HIV-1 protease (**Figure 2**). We reasoned that although the zymogen design satisfied the steric requirements as deduced by the high yield of *in vitro* refolded protein, the accessibility of the protease might somehow be impeded. Consequently, ONCYPG1, ONCYPG2 and ONCYPGG were designed to elongate the linker and increase its flexibility in order to facilitate the protease recognition and catalysis. On the other hand, Beck and co-workers [28] had reported that the peptide substrate GSGIF*LETSL identified from a phage display library, was cleaved 60 times more efficiently than the MA/CA-based control phage with the sequence SGVSQNY*PIVQVL. Therefore, two additional variants, ONCFL and ONCFLG, were constructed to assay this alternative recognition sequence. When comparing the activation of the different variants as a function of the linker length, it was revealed that the longer the linker, the more efficient was the cleavage by the protease (**Figure 2B**). As shown in the Results section, the new variants comprising the Y*P cleavage site only improved the final yield to 3–4 fold, compared to ONCYP, while ONCFL and ONCFLG were successfully cleaved by the protease (**Figure 2A and 2B**). Besides, the ONCFLG variant is more rapidly cleaved than ONCFL which could be explained by a facilitated accessibility of the protease to the ONCFLG scissile bond due to the additional Gly residue (**Figure S3**). Thus, the differences in the cleavage yields among the ONC variants are similar to the results for different HIV-1 protease substrates reported previously [**28**]. Therefore, the affinity for a particular amino acid sequence seems to be the main reason underlying the differences in cleavage observed between the YP- and FL-variants. Nevertheless, other factors such as interaction of the linker with the remainder of the protein, the linkeŕs conformation and the orientation of the residues near the scissile bond could also affect the final efficiency of the variant cleavage although in a lesser extent.

To be effective, the intact ONC zymogens should have low enzymatic activity before activation and a substantial increase in the catalytic efficiency should be observed after cleavage by the protease. Here, the enzymatic activity and cytotoxicity of the variants was determined only for those that could be completely cleaved, ONCFL and ONCFLG. Both the ONCFL and ONCFLG uncleaved variants showed low ribonucleolytic activity and slight cytotoxicity against human T-lymphocytes Jurkat cells which are desirable characteristics of a zymogen. However, only a minor increase in the catalytic activity of 1.27- and 1.16-fold for ONCFL and ONCFLG, respectively, is observed following HIV-1 protease cleavage. In addition, in these initial studies we have shown by confocal laser scanning microscopy that Alexa-labeled ONCFLG efficiently penetrates Jurkat cells. To better understand the cleavage process and explain the low increase in activity following cleavage, we solved the 3D structure of ONCFLG zymogen by heteronuclear NMR spectroscopy. The well-defined structure we obtained is closely similar to that of wild type ONC, except the linker segment Gly33-Gly48 does not adopt any regular secondary structure and shows high structural diversity. In addition, based on the measurement of heteronuclear NOEs, this linker segment is also the most dynamic region of the molecule in the uncleaved form. The conformational flexibility exhibited by the linker, together with the accessibility of the Phe40-Leu41 residues that contain the scissile bond (**Figure S3**), explains the rapid cleavage of ONCFLG variant. After cleavage, the new termini show NOE values characteristic of unstructured peptides with a high degree of conformational freedom.

The residue-level hydrogen exchange measurements corroborate the extraordinary stability of ONCFLG as determined by DSC. The remarkable stability of the ONC variants is somewhat surprising considering that the N- and C-termini have been permuted. No measurable protection against ^1^H-^2^H exchange was observed for the intact or cleaved linker segment; this is consistent with the lack of well-defined secondary structure and highly dynamic behavior indicated other NMR results. Upon cleavage, the ΔG_HX_ values throughout the protein become slightly lower on average which suggests that interactions between the linker and the protein core make modest contributions to the latter’s stability.

Comparison of the ONCFLG structure with the available wild type ONC structures also sheds light on the variation in ribonucleolytic activity observed between the uncleaved and cleaved forms. Although there are no differences in occupancy for residues belonging to the B1 and B2 subsites, remarkable changes are observed for the catalytic residues. Raines and co-workers [13] showed that the Pyr1 to Ala substitution resulted in a 20-fold decrease in ONC’s ribonucleolytic activity and suggested that the loss of the Pyr1 Oε-Lys9 Nξ hydrogen bond would provide Lys9 with rotational freedom, reducing catalysis. In addition, replacing either Lys9 or Lys31 with an Ala decreased the value of *k*_cat_/K_M_ by 10^3^-fold. The catalytic activity of the double mutant was below the sensitivity limit of the assay. These results lead to the conclusion that both Lys9 and Lys31 contribute to the stabilization of the transition state. This ONCFLG structure reveals that the loss of this hydrogen bond is responsible for the conformational freedom observed for Lys57 side chain (Lys9 in wild type ONC) and corroborates that the function of Pyr1 in wild type ONC is to position Lys9 appropriately for catalysis. More precisely the Lys79 side chain (Lys31 in wild type ONC) turns away from the main body of ONCFLG (**Figure 4A**). In ONCFLG, the mobility of His58 increases and the imidazole ring of His25 can rotate about the χ_2_ dihedral angle without adopting the A or B conformations observed in the ONC-d(AUGA) complex (2I5S) and in the wild type ONC (1ONC) structure, respectively (**Figure 4B and 4C**). These changes are likely to be consequences of the movements of Lys57 and the presence of the bridge that connects the native N- and C- termini. The catalytic efficiency of the ONCQ1S variant, that also likely lacks the aforementioned hydrogen bond between Pyr1 Oε and Lys57 Nξ, is about 3-fold lower than that of wild type ONC while the k_cat_/K_M_ values of ONCFL and ONCFLG are 20- and 7-fold lower, respectively. Therefore, the intact linker directly contributes to lower the catalytic efficiency of ONCFLG variant by 2-fold and that of ONCFL to 6-fold, approximately. Taken together, all these structural characteristics account reasonably well for ONCFLG´s low ribonucleolytic activity.

The structural model for the cleaved ONCFLG (**Figure 5**) reveals that the linker segments may adopt a higher number of conformations following hydrolysis. The movement of these segments could sterically obstruct the substrate’s approach to the active site, increase the K_M_, and worsen the already poor nucleotide-binding affinity that has been proposed to account in large measure for the low ribonucleolytic efficiency of ONC [13], [31]. This effect, together with the alterations in the active site residues in the uncleaved form described above, most of which remain in the cleaved form, could be the basis of the low catalytic activities of the cleaved circularly permuted ONC variants and explain why their ribonucleolytic activities are not as high as that of ONCQ1S.

### Conclusions

In this work we have designed and optimized circularly permuted ONC variants that are efficiently cleaved by the HIV-1 protease. However, properly cleaved ONC-FL variants showed marginal activation and both activity and cytotoxicity were diminished due to circular permutation. Also, we have solved the three-dimensional structure of ONCFLG. The structural data reported herein for ONCFLG lay the groundwork for the future optimization of zymogens via the redesign of linker length and sequence to better position the hydrogen bond acceptor group to fix the Nξ of Lys57, insertion of additional substitutions such as Met28Leu [40] aimed at increasing the catalytic efficiency or, the design of additional protease cleavage sites within the linker to diminish the steric hindrance of the segments following cleavage. Therefore, the creation of successful zymogens based on ONC would represent a new versatile option to manage the enzymatic activity of this enzyme and target its toxicity to a cell in a specific diseased state. ONC-based zymogens activated by microbial or viral proteases could extend the therapeutic utility of ONC to maladies other than cancer and represent a strategy to avoid known mechanisms of pathogenic resistance.

## Supporting Information

Figure S1 **Internalization of ONCFLG-Cys variant**. Uncleaved ONCFLG-Cys variant, (2 µM) labeled with Alexa 488, was incubated with Jurkat cells for known times, and cells were then washed with PBS three times prior visualization. In all samples, cell nuclei and membranes were counterstained with Hoechst and DiI, respectively, for 10 min before washing. Internalization was visualized with a Leica TCS SP2 AOBS laser scanning confocal microscope.
(TIF)

Figure S2 **Comparison of homologous disulfide bonds from** **ONCFLG variant and wild type ONC.** Comparison of homologous Cys18-Cys96 and Cys48-Cys90 disulfide bonds connecting β-strand β6 and α-helix α3 and Cys15-Cys32 and Cys87-Cys104 disulfide bonds connecting β-strand β6 and β-strand β7 in ONCFLG variant and wild type ONC (1ONC.pdb), respectively. Different conformations from ONCFLG variant Cys18-Cys96 and Cys15-Cys32 disulfides are shown in CPK colors and wild type Cys48-Cys90 and Cys87-Cys104 disulfides are shown in magenta. This representation was generated using VMD [41].
(TIF)

Figure S3 **Structure of the ONCFLG variant showing the linker region, active site residues and disulfide bonds.** The different conformations of the linker region Gly33-Gly38 are shown in orange while the rest of the molecule is shown in grey ribbon. Phe40-Leu41, that constitute the scissile bond are shown in red, Ser49, Lys57 and Phe26 are colored in blue and His58, Lys79 and His25 are in purple. This representation was generated using VMD [41].
(TIF)

Table S1 **Theoretical and experimental molecular masses of the proteins used in this work.**
(DOCX)

Supporting Information S1 **Includes supplementary materials and methods, results and the corresponding bibliography.**
(PDF)
