# Supplementary figures and images for: Towards Tricking a Pathogen’s Protease into Fighting Infection: The 3D Structure of a Stable Circularly Permuted Onconase Variant Cleavedby HIV-1 Protease
Source: PLoS One. 2013 Jan 18;8(1):e54568. doi: 10.1371/journal.pone.0054568 (PMC3548804; doi:10.1371/journal.pone.0054568)

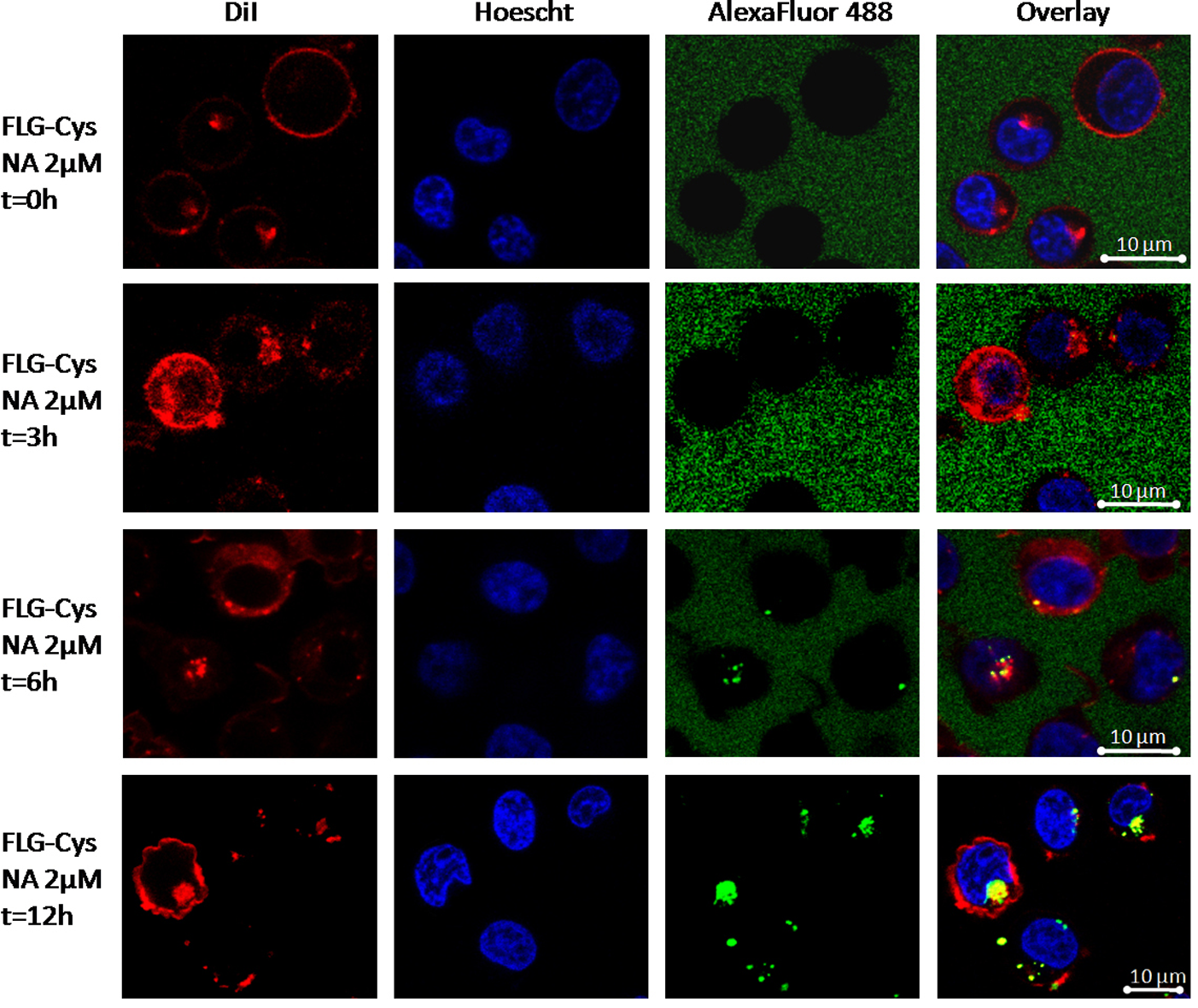

Supplement: Figure S1 — Internalization of ONCFLG-Cys variant. Uncleaved ONCFLG-Cys variant, (2 µM) labeled with Alexa 488, was incubated with Jurkat cells for known times, and cells were then washed with PBS three times prior visualization. In all samples, cell nuclei and membranes were counterstained with Hoechst and DiI, respectively, for 10 min before washing. Internalization was visualized with a Leica TCS SP2 AOBS laser scanning confocal microscope. (TIF) [file pone.0054568.s001.tif]

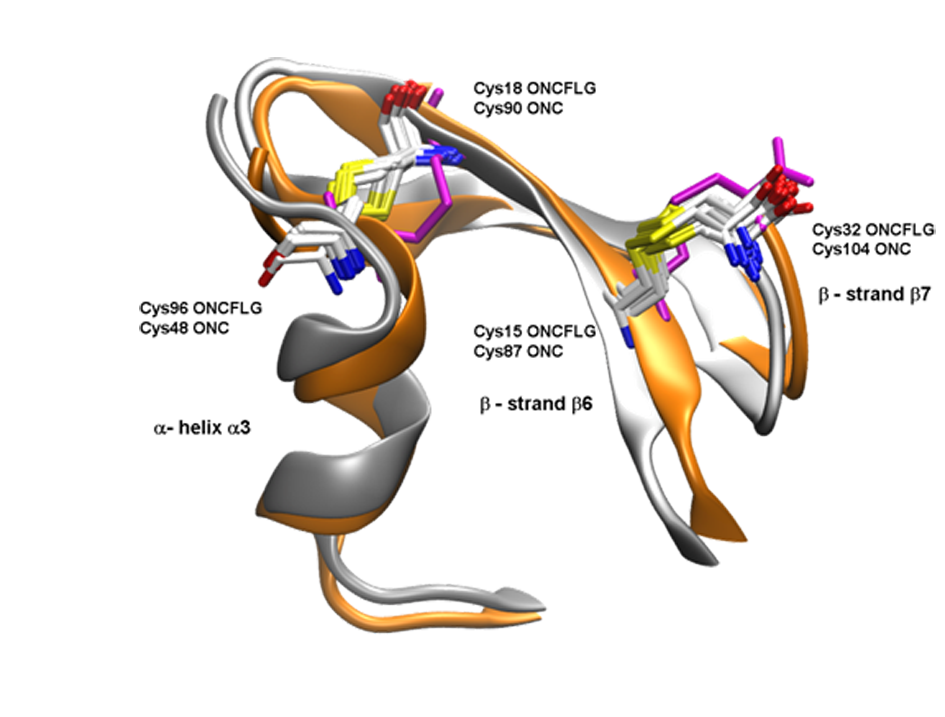

Supplement: Figure S2 — Comparison of homologous disulfide bonds from ONCFLG variant and wild type ONC. Comparison of homologous Cys18-Cys96 and Cys48-Cys90 disulfide bonds connecting β-strand β6 and α-helix α3 and Cys15-Cys32 and Cys87-Cys104 disulfide bonds connecting β-strand β6 and β-strand β7 in ONCFLG variant and wild type ONC (1ONC.pdb), respectively. Different conformations from ONCFLG variant Cys18-Cys96 and Cys15-Cys32 disulfides are shown in CPK colors and wild type Cys48-Cys90 and Cys87-Cys104 disulfides are shown in magenta. This representation was generated using VMD [41]. (TIF) [file pone.0054568.s002.tif]

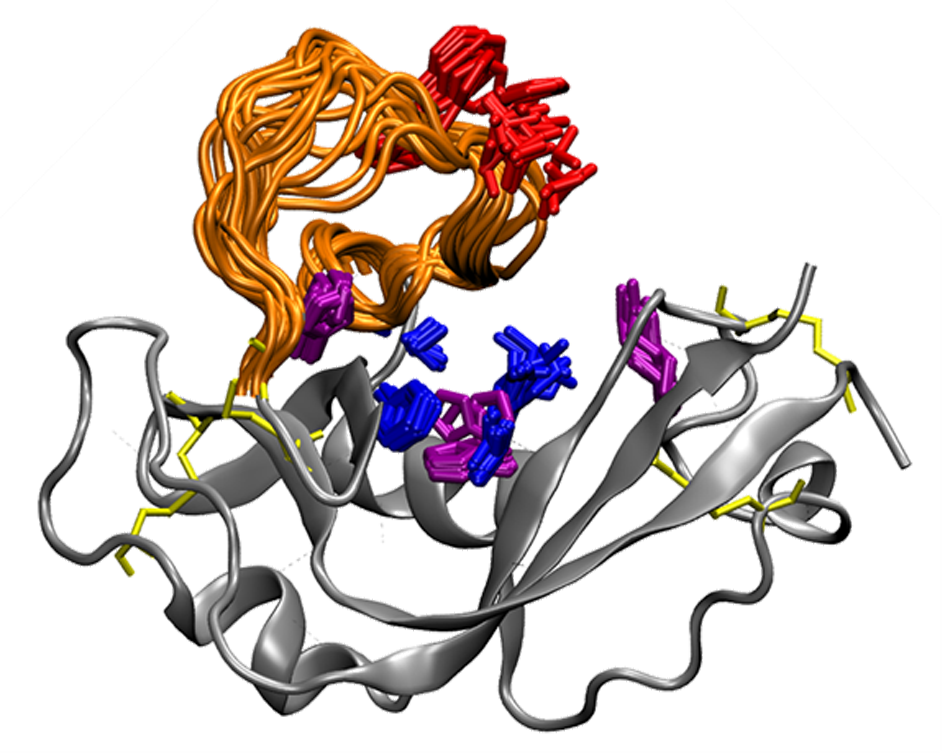

Supplement: Figure S3 — Structure of the ONCFLG variant showing the linker region, active site residues and disulfide bonds. The different conformations of the linker region Gly33-Gly38 are shown in orange while the rest of the molecule is shown in grey ribbon. Phe40-Leu41, that constitute the scissile bond are shown in red, Ser49, Lys57 and Phe26 are colored in blue and His58, Lys79 and His25 are in purple. This representation was generated using VMD [41]. (TIF) [file pone.0054568.s003.tif]
