# Supplementary material for: Towards Tricking a Pathogen’s Protease into Fighting Infection: The 3D Structure of a Stable Circularly Permuted Onconase Variant Cleavedby HIV-1 Protease
Source: PLoS One. 2013 Jan 18;8(1):e54568. doi: 10.1371/journal.pone.0054568 (PMC3548804; doi:10.1371/journal.pone.0054568)
Supplement: Table S1 — Theoretical and experimental molecular masses of the proteins used in this work. (DOCX) [file pone.0054568.s004.docx]

**Table S1. Theoretical and experimental molecular masses of the proteins used in this work.**

| Protein | Theoretical molecular mass (Da)^a^ | Observed molecular mass (Da)^b^ |
| --- | --- | --- |
| HIV-1 protease | 10792.79 | 10793.27 |
| ONC | 11819.84 | 11818.46 |
| ONCQ1S | 11803.78 | 11804.27 |
| ONCFLG-Cys | 13364.37 | 13363.28 |
| ONCYP | 13273.20 | 13274.07 |
| ONCYPG1 | 13330.26 | 13330.16 |
| ONCYPG2 | 13330.26 | 13329.73 |
| ONCYPGG | 13387.31 | 13389.43 |
| ONCFL | 13291.26 | 13295.54 |
| ONCFLG | 13348.31 | 13348.61 |

^a^ Theoretical molecular masses were calculated using the Compute pI/Mw tool from the ExPASy Bioinformatics Resource Portal, <http://web.expasy.org/compute_pi/> (Swiss Institute of Bioinformatics).

^b^ Molecular masses were confirmed by matrix-assisted laser desorption ionization time-of-flight (MALDI-TOF) mass spectrometry using a Bruker-Biflex equipment at the Servei de Proteómica de la UCTS de l’Institut de Recerca de l’Hospital Universitari Vall d’Hebron, Barcelona (Spain).
