# Supplementary material for: Towards Tricking a Pathogen’s Protease into Fighting Infection: The 3D Structure of a Stable Circularly Permuted Onconase Variant Cleavedby HIV-1 Protease
Source: PLoS One. 2013 Jan 18;8(1):e54568. doi: 10.1371/journal.pone.0054568 (PMC3548804; doi:10.1371/journal.pone.0054568)
Supplement: Supporting Information S1 — Includes supplementary materials and methods, results and the corresponding bibliography. (PDF) [file pone.0054568.s005.pdf]

Supporting Information S1

Towards Tricking a Pathogen´s Protease into Fighting Infection: the 3D Structure of a Stable Circularly Permuted Onconase Variant Cleaved-by HIV-1 Protease

Callís, M., et al.

Contents:

Supplementary Materials and Methods

Sequences of Oligos used for the constructs

Enzymatic Activity of HIV-protease

Supplementary Results

Interactions of the d(UGG)$_3$ substrate analog with *ONCFLG* variant.

Supplementary References

SUPPLEMENTARY MATERIALS AND METHODS

Materials

The *E. coli* strains BL21(DE3), Rosetta^{TM} 2(DE3) and the ribonuclease substrate 6-FAM-dArUdAdA-6-TAMRA were obtained from Novagen (Madison, WI, USA). The nonhydrolyzable substrate analog 5'd[UGGUGGUGG]3' (abbreviated as d(UGG)$_3$ was purchased from Thermal-Fischer (Ulm, Germany). Oligonucleotides used for site-directed mutagenesis and molecular biology enzymes were from Roche (Basel, Switzerland). Other chemicals were from Sigma (ST. Louis, MO, USA).

The Mono-S HR 5/5 column was from Amersham Biosciences (Piscataway, NJ, USA). Cell lines were provided by American Type Culture Collection (Manassas, VA, USA). RPMI GlutaMAX™-I medium as well as penicillin and streptomycin were

provided by Gibco^{RTM} (Invitrogen Life Sciences, Carlsbad, CA, USA). Fetal clone II serum (FBS) was purchased from Hyclone (Logan, UT, USA).

Design of circularly permuted ONC variants to get a zymogen

To generate a structural model of the ONC zymogens, the atomic coordinates of wild type ONC were obtained from the Protein Data Bank (pdb accession code 1ONC) [2]. After *in silico* mutation of Gln1 to Ser1 using Swiss-PdbViewer, a new pdb file was created. Using a text processor the atomic coordinates from Arg73 to Cys104 were cut and pasted just before the remaining ONC atomic coordinates (from Ser1 to Ser72). The new file was opened with Swiss-PdbViewer so that it could be used as a template. A raw sequence in FASTA format corresponding to the desired zymogen sequence was loaded and manually aligned to the sequence of the pdb template. Finally SWISS-MODEL, an automated homology modeling server, was utilized to generate the model and optimize it by 3000 steps of steepest gradient energy minimization using the GROMOS 96 implementation of Swiss-PdbViewer [3]. When using linkers shorter than 14 residues, the retrieved models were either distorted because there were too many contacts between the linker and the main body of the protein or the final energy of the model after minimization was too high, i.e. positive. Therefore, those models with less than 14 residues were considered to be poor candidates for developing onconase zymogens.

Plasmid construction

All the plasmids coding for ONC variants and zymogens or circularly permuted variants were obtained using pONC [4] as template. For the construction of pONCQ1S, the gene coding for ONC was amplified with oligos 5'-gcccagccggcgatggccagcgactggctgac-3' and the T7 terminator. The product was purified

and digested with *Mlu*NI (underlined) and *Sal*I and inserted into pET22 b (+) (Novagen, Madison, WI, USA).

Plasmids that direct the production of ONC zymogens were constructed by circular permutation following the strategy described by Raines and coworkers [5]. Accordingly, oligos T7 promoter and 5'-cccccccccccggatcc**gca**agaaccaacaccaac-3' were used to amplify the first copy of ONC from pONC, remove the stop codon and insert a *BamH*I site (underlined) after the Cys104 codon (bold). The amplification product was digested with *Nde*I and *BamH*I and ligated into pET22 b (+) to yield pONC_NdeIBamHI. The second ONC gene copy was also amplified from pONC using oligos 5'-cccccccccggccggc**tcc**gactggctgactttcc-3' and the T7 terminator to insert an *EclX*I site (underlined) and to substitute Gln1 for Ser (bold). The amplified product was purified and digested with restriction endonucleases *EclX*I and *Sal*I and ligated into pONC_NdeIBamHI to yield pBONC.

*BamH*I and *EclX*I sites in pBONC were used to insert the desired DNA cassettes coding for the amino acid sequences that should link the native N- and C-termini while containing the HIV1 cleavage site. The plasmids containing the two copies of ONC gene in tandem connected by the linkers were named pBONCYP, pBONCYPG1, pBONCYPG2, pBONCYPGG, pBONCFL and pBONCFLG. The different cassettes and the encoded amino acid sequences are listed in the following table.

Amino acid sequences with a built-in cleavage site for HIV-1 protease and the corresponding oligonucleotide cassettes.

plasmid/zymogen	linker sequences [a]	cassette oligos
pONCYP/ONCYP	**SC$_{104}$**GSGSQNY*PIVQSAG**S$_1$D**	5’-gatccggttctcagaactacccgatcgttcagtc-3’<br>5’-ggccgactgaacgatcgggtagttctgggaaccg-3’
pONCYPG1/ONCYPG1	**SC$_{104}$**GSGGSQNY*PIVQSAG**S$_1$D**	5’-gatccggtggttcccagaactacccgatcgttcagtc-3’<br>5’-ggccgactgaacgatcgggtagttctgggaaccaccg-3’
pONCYPG2/ONCYPG2	**SC$_{104}$**GSGSQNY*PIVQGSAG**S$_1$D**	5’-gatccggttcccagaactacccgatcgttcagggttc-3’<br>5’-ggccgaaccctgaacgatcgggtagttctgggaaccg-3’
pONCYPGG/ONCYPGG	**SC$_{104}$**GSGGSQNY*PIVQGSAG**S$_1$D**	5’-gatccggtggttcccagaactacccgatcgttcagggttc-3’<br>5’-ggccgaaccctgaacgatcgggtagttctgggaaccaccg-3’
pONCFL/ONCFL	**SC$_{104}$**GSGSGIF*LETSLSAG**S$_1$D**	5’-gatccggttccggtatcttcctggaaacctccctgtc-3’<br>5’-ggccgacagggaggtttccaggaagataccggaaccg-3’
pONCFLG/ONCFLG	**SC$_{104}$**GSGGSGIF*LETSLSAG**S$_1$D**	5’-gatccggtggttccggtatcttcctggaaacctccctgtc-3’<br>5’-ggccgacagggaggtttccaggaagataccggaaccaccg-3’

[a] The residues belonging to the C- and N-termini of ONC are indicated in bold and the HIV-1 protease recognition sequences are underlined. Residues separated by an asterisk constitute the S_1 and S_1’ subsites of the protease.

The permuted ONC with Arg73/Ser72 termini containing the different linkers were created by PCR using the above mentioned plasmids as templates and oligos 5’-ctgactgcaacgttcatatgcgtccgtgcaaatac-3’ and 5’-cagtttgtattttgtcgactaagaagtaacgttgc-3’ with the *Nde*I and *Sal*I restriction sites underlined, respectively. The purified PCR product were digested with *Nde*I and *Sal*I endonucleases and ligated to previously *Nde*I- and *Sal*I-digested pET22b(+) to yield plasmids pONCYP, pONCYPG1, pONCYPG2,

pONCYPGG, pONCFL and pONCFLG that direct the production of the different ONC variants.

The ONCFLG-Cys variant was designed to monitor its internalization after its conjugation to Alexa Fluor® 488 C5 maleimide (Molecular Probes, Life Technologies, Carlsbad, CA, USA). The plasmid used to express this variant was constructed using pONCFLG as a template by site-directed mutagenesis using the QuikChange™ kit from Stratagene (La Jolla, CA). Synthetic oligonucleotides 5’-tctgactgcaacgttacttgctagtcgagcaccacc-3’ and 5’-ggtggtgctcgactagcaagtaacgttgcagtcaga-3’ were used to substitute the C-terminal Ser of ONCFLG for Cys (underlined together with the stop codon).

The plasmid pSK#1 that directs the production of HIV-1 protease was constructed by amplification of the full length HIV-1 protease gene plus eight additional residues (MGTVSNF) encoded upstream of the protease-coding region, from plasmid pSK616 (a kind gift of Dr. A. Villaverde from the Universitat Autònoma de Barcelona). The oligos used for the HIV-1 protease gene amplification, 5’-cccccccccatatgggaactgtatccttt-3’ and 5’-cccccccctgtcgactaaaaatttaaagtgc-3’, included *Nde*I and *Sal*I sites (underlined), respectively, which were designed to clone the protease-coding DNA into the equivalent sites of pET22b(+).

Production of circularly permuted ONC variants, ONCQ1S, wild type ONC and HIV-1 protease.

Circularly permuted ONC variants, ONC and ONCQ1S were produced and purified essentially as described previously for wild type ONC [6,7]. Briefly, BL21 (DE3) cells transformed with the corresponding plasmid were grown until an OD_{550} of

1.5 was reached. Protein expression was induced by adding isopropylthiogalactoside (IPTG) to 1 mM. After 3-4 h, the cells were collected by centrifugation, lyzed with a French Press set at 1100 psi, and inclusion bodies were harvested by centrifugation. The pellets were then resuspended in 10 mL of 6 M guanidinium chloride, 2 mM EDTA, 100 mM Tris-acetate, pH 8.5, to assist protein solubilization. Samples were reduced by the addition of reduced glutathione (GSH^{red}) to a final concentration of 0.1 M, pH adjusted to 8.5 with solid Tris, and then the samples were incubated at room temperature for 2 h under nitrogen atmosphere. Insoluble material was then removed by centrifugation (12000*xg*, 30 min, 4 ºC). Solubilized and reduced proteins were diluted drop-wise (≈100-fold), to a final concentration of 50–100 $\mu g \cdot mL^{-1}$, into 0.5 M L-arginine, 1 mM oxidized glutathione ($GSSG^{ox}$), 2 mM EDTA, 0.1 M Tris-acetate, pH 8.5, and then incubated at 10 ºC for at least 48 h. To stop oxidation, the pH was adjusted to 5.0 with acetic acid. The refolded samples were then concentrated by ultrafiltration using a Prep/scale TFF cartridge (Millipore, Bedford, MA). Wild type ONC was additionally dialyzed against 200 mM sodium phosphate, pH 7.2, for 48 h at room temperature, to allow for the cyclization of N-terminal glutamine to pyroglutamic acid. Precipitated or insoluble material was eliminated by centrifugation (12000 *g*, 10 min, 4 ºC). Refolded samples were then loaded onto a Mono-S HR 5/5 FPLC column (Amersham Biosciences, Uppsala, Sweden) and eluted with a linear gradient of 0–600 mM NaCl in 30 min.

For the production of HIV-1 protease, 25 mL of LB media supplemented with 50 μg/mL of ampicillin and 35 μg/mL cloramphenicol, were inoculated with a single colony of *E.coli* Rosetta™ 2(DE3) transformed with plasmid pSK#1 carrying the full length HIV-1 protease plus encoded eight additional residues (MGTVSNF) at the N-terminus.

After 5 hours with agitation at 37°C, the starting culture was used to inoculate 1/100 (v/v) 2 L of LB media supplemented with the antibiotics previously stated. When the culture reached an OD_{550nm} of 1.5-2.0 OD, HIV-1 protease expression was induced by the addition of IPTG (1 mM final concentration). Four hours later, cells were collected by centrifugation, lyzed with a French Press set at 1100 psi, and inclusion bodies were harvested by centrifugation. The inclusion bodies were dissolved in 20 mM Tris-acetate, pH 8.0, containing 8 M urea and 10 mM DTT to a final concentration of 10 mg/mL. The solution was incubated with gentle agitation for 2 hours. The insoluble debris was removed by centrifugation at 12000*xg* for 30 min at 4 ºC. The supernatant was loaded onto a column containing 50 mL of DEAE resin (Bio-Rad, USA) previously equilibrated with the solubilization buffer. The flow-through fractions were dialyzed overnight at 4 °C against 100 mM NaH_2PO_4, pH 6.7, 0.01% CHAPS, 1 mM EDTA, 1 mM DTT and 25 % (v/v) glycerol. Afterwards, the sample was dialyzed against 20 mM NaH_2PO_4, pH 6.7, 1 mM EDTA, 1 mM DTT and 1 % (v/v) glycerol prior to the final purification using a Mono-S HR 5/5 FPLC column. HIV-1 protease was eluted using a linear NaCl (0-400 mM) gradient in buffer containing 20 mM NaH_2PO_4, pH 6.7, 1 mM EDTA, 1 mM DTT and 1 % (v/v) glycerol. 10% glycerol was added to the protease containing fractions which were stored at - 80°C until use.

Protein concentrations were determined using the Bradford assay [8]. Protein purity and homogeneity were confirmed by SDS-PAGE. Molecular masses were confirmed by matrix-assisted laser desorption ionization time-of-flight (MALDI-TOF) mass spectrometry using Bruker-Biflex equipment at the Servei de Proteómica de la

UCTS de l'Institut de Recerca de l'Hospital Universitari Vall d'Hebron, Barcelona (Spain).

Kinetic characterization of recombinant HIV-1 protease

A fluorescence resonance energy transfer (FRET) assay [9] was used to characterize the enzymatic activity of the HIV-1 protease produced in this work and compare it with commercial recombinant HIV-1 protease purchased from Bachem (Switzerland). The quenched fluorogenic peptide DABCYL-γ-Abu-Ser-Gln-Asn-Tyr*Pro-Ile-Val-Gln-EDANS (Bachem, Switzerland) contains the octapeptide sequence SQNY*PIVQ which corresponds to the naturally occurring Pr55gag p17/p24 HIV-1 cleavage site where Tyr*Pro is the scissile peptide bond. Standard assays were performed in 0.1 M sodium acetate, 1 M NaCl, 1 mM EDTA, 1 mM DTT, 10% DMSO, 1 mg/mL BSA, pH 4.7 in a final volume of 120 μL, at 30°C. Substrate concentrations ranged from 0 to 130 μM and the protease concentration was 1.45 nM. Cleavage of the substrate results in an increase in fluorescence intensity (excitation at 335 nm; emission at 470 nm) which was monitored in one second steps for five min after the protease addition using a thermostated Lambda LS50 fluorescence spectrometer (Perkin-Elmer, Waltham, MA, USA). The rates of hydrolysis of the substrate ($Vobs_{FI}$ in fluorescence units/s) for both proteases and for each substrate concentration were determined by triplicate.

The inner filter effect (IFE) was corrected and the observed rates ($Vobs_{FI}$) converted to V (μM min^{-1}) as previously described [10]. Finally, data was fitted to the Michaelis-Menten equation and the kinetic parameters V_{max} and K_M determined for both commercial recHIV-1 PR and that produced in our laboratory.

Kinetic parameters of HIV-1 protease produced in this work compared to those of the commercially available recHIV-1 PR (Bachem, Switzerland)

	V_{max} (µM min^{-1})^a	K_M (µM)^a	k_{cat}/K_M (µM^{-1}/min^{-1})	Relative catalytic efficiency (%)
HIV-1 protease	0.174±0.003	14.04±0.80	17.10±0.98	90
Commercial HIV-1 PR	0.150±0.010	10.94±1.020	18.91±1.23	100

[a] V_{max} and K_M were determined for HIV-1 protease using DABCYL-γ-Abu-Ser-Gln-Asn-Tyr*Pro-Ile-Val-Gln-EDANS (Bachem, Switzerland) as a substrate. Assays were performed in 0.1 M sodium acetate, 1M NaCl, 1mM EDTA, 1 mM DTT, 10% DMSO, 1 mg/mL BSA, and pH 4.7 in a final volume of 120 µL, at 30°C. Substrate concentrations ranged from 0 to 130 µM and protease concentration was at 1.45 nM.

Calorimetric conformational stability determination

The stability of wild type ONC, ONCQ1S, ONCFL and ONCFLG variants was determined by differential scanning calorimetry (DSC) at the Plataforma de Polimorfisme i Calorimetria from the Serveis Cientificotècnics of the Universitat de Barcelona by means of a VP-DSC apparatus from MicroCal (Northampton, MA, USA). For these measurements, proteins were dissolved in 0.1 M 2-[N-morpholine] ethanesulfonic acid-NaOH (MES-NaOH) buffer, pH 6.0 at concentrations of 0.15-0.18 µM as determined by Bradford assay [8], and temperature was increased from 30 to 110 ºC using a scan rate of 1 K min^{-1}. T_d is the denaturation temperature and corresponds to the maximum of the DSC peak.

Circularly permuted ONC variants activation

The activation of the different circularly permuted ONC variants was assayed by mixing them with different molar equivalents of the HIV-1 protease (from 1:5 to 1:100 protease:zymogen ratios), in buffer composed of 100 mM sodium acetate, pH 4.7, 300 mM NaCl and 4 mM EDTA. Aliquots were taken at different times, mixed with SDS-PAGE loading buffer and stored at -20° until subjected to electrophoresis. Progress of the activation reactions was quantified using the Imaging System FluorChem® SP (Alpha Innotech, San Leandro, CA, USA).

Enzymatic activity of circularly permuted ONC variants

The ribonucleolytic activity of the circularly permuted ONC variants before and after cleavage was measured with the fluorogenic substrate 6-FAM-dArUdAdA-6-TAMRA [11](Kelemen et al., 1999; Park et al., 2001) using a thermostated Lambda LS50 fluorescence spectrometer (Perkin-Elmer, Waltham, MA, USA) equipped with sample stirring. Cleavage of this substrate results in an increase in fluorescence intensity (excitation at 492 nm; emission at 515 nm). Assays were performed at 25ºC in 1.5 ml of 0.1 M MES-NaOH buffer, pH 6.0, containing 0.1 M NaCl, 50 nM 6-FAM-dArUdAdA-6-TAMRA and 15 μM wild type ONC and 50 μM circularly permuted ONC variants. According to [12], data were fit to the equation:

$$k_{cat}/K_m = (\Delta F/\Delta t) / \{(F_{max} - F_0)\ [E]\}$$

where $\Delta F/\Delta t$ is the initial rate of the reaction, F_0 is the initial fluorescence intensity prior to the addition of the enzyme, F_{max} is the fluorescence intensity after complete cleavage of the substrate, obtained following the addition of wild type RNase A, and [E] is the

ribonuclease concentration. Each measurement was repeated at least three times and values of **Table 1** in the main text are expressed as a mean ±SD.

Cytotoxicity of circularly permuted ONC variants

Cells were grown in RPMI GlutaMAX™-I media supplemented with 10% FBS and 1% penicillin/streptomycin. The cytotoxicity of wild type ONC, ONCQ1S and circularly permuted ONC variants was assayed on human T-lymphocytes Jurkat cells which were seeded into 96-well plates at the appropriate density (6000 cells/well). Cells were incubated with different concentrations of the proteins ranging from 0.1 to 30 μM. After three days, the cytotoxicity of onconases was measured using an assay that monitors the reduction of (3-(4,5-dimethylthiazol-2-yl)-2,5-diphenyltetrazolium bromide (MTT) to formazan (Celltiter 96® Aqueous, Promega, Madison, WI, USA) essentially as described by the manufacturer instructions. Three hours after the addition of MTT, the absorbance was read at 570 nm using an Elx800 microplate reader (Bio-Tek Instruments, Inc., Winooski, VE, USA). The results for a single experiment are the average of three determinations, and the experiments were repeated independently three times each. The IC_{50} values represent the concentration of the assayed enzyme required to inhibit cell proliferation by 50%.

Internalization of ONCFLG-Cys variant

A Cys residue was introduced by site-directed mutagenesis as described above at the C-terminus of ONCFLG variant to allow fluorochrome conjugation. ONCFLG-Cys was reacted with Alexa Fluor® 488 C5 maleimide (Molecular Probes, Life Technologies, Carlsbad, CA, USA) essentially as recommended by the manufacturer. Briefly, 100 μM ONCFLG-Cys in PBS buffer was incubated with a 10-fold molar excess of DTT for 1 h

in N_2 atmosphere. Excess DTT was removed by gel filtration on a PD10 column. A 10-fold molar excess of reactive dye was immediately added to the protein solution. The reaction was stopped after overnight incubation in dark conditions at 4°C by adding 1 mM reduced glutathione, passed through a PD10 gel-filtration column, filter-sterilized, and stored at -20 °C. The maleimide reaction on the Cys residue generates a thioether bond which is considered to be irreversible within animal cells.

Jurkat cells were grown in RPMI GlutaMAX™-I media supplemented with 10% FBS and 1% penicillin/streptomycin. The day before protein incubation, cells were seeded onto 6- or 24-well tissue culture dishes pretreated with poly-lysine to yield 75% confluency the next day. Protein was incubated with cells for known times, and cells were then washed with PBS three times prior visualization. In all samples, cell nuclei and membranes were counterstained with Hoechst 33342 (Molecular Probes, Eugene, OR, USA) and DiI, respectively, for 10 min before washing. Internalization was visualized with a Leica TCS SP2 AOBS laser scanning confocal microscope equipped with a HCX PL APO 63x1.4 oil objective at the Servei de Microscopia de la Universitat Autònoma de Barcelona. Images were acquired as 512 x 512 pixel images. All images were taken on the same day using the same laser intensity. Images were taken as 3 x 3 in sections from the original file and reduced by 50% for publication.

NMR experiments: structure, dynamics and conformational stability

Uniformly doubly labeled ^{13}C, ^{15}N recombinant ONCFLG variant, was produced, purified from *E. coli* BL21(DE3) cells, and used for NMR experiments, essentially as described previously [6] [13].

For the structure calculation, NOE assignments were obtained from 2D ^{1}H-^{1}H NOESY with 80 ms mixing time, and from 3D ^{15}N-NOESY-HSQC, ^{13}C-NOESY-HSQC (aliphatic), ^{13}C-NOESY-HSQC (aromatic) spectra, with 50 ms mixing times. In order to characterize the backbone dynamics, ^{15}N-^{1}H NOE experiments were recorded with a relaxation delay of 5.0 s and analyzed as described [14].

The exchange of amide protons with solvent deuterons was done at 30 ºC and the pH was 6.0 for the uncleaved ONCFLG form and 5.2 for the cleaved form. The hydrogen exchange rates were determined from a series of consecutively HSQC spectra following a dead time of about 30 min. The intrinsic exchange rates for fully denatured proteins were calculated using the parameters reported by [15] corrected for pH and temperature. The conformational stability, ΔG_{HX}, of each amide group was calculated from the protection factor as described [16].

All the NMR spectra were recorded on a Bruker AV 800 MHz spectrometer equipped with a cryoprobe, processed using TopSpin (Bruker BioSpin, Rheinstetten, Germany) and analyzed with SPARKY [17]. MolMol [18] and VMD [1] were used to display NMR structures.

Structure calculation

The structure calculation was performed with CYANA [19] using the automatic NOE assignment facility combined with a manual check of all assigned NOEs. Backbone dihedral angle constraints were determined from chemical shift values using TALOS+ [20].

Initially 100 conformers were generated that were forced to satisfy experimental data during a standard automatic CYANA protocol. The 20 conformers with the lowest

final CYANA target function values were selected and subjected to 2000 steps of energy minimization with AMBER9 [21]. The final structure quality was checked with PROCHECK-NMR [22] and the coordinates have been deposited in the PDB under the accession number 2LT5. Statistics of the calculation are summarized in **Table 2** in the main text.

Structural model of the cleaved ONCFLG form

The model of cleaved ONCFLG form was computed with the program CYANA [19] according to the NMR evidence indicating that the global fold is conserved. We have used the complete list of distance and angular restrictions for residues 1-32 and 49-120 obtained for the uncleaved form as described, and we have discarded those intra- and inter-residue constraints corresponding to the disordered Gly33-Phe40 and Leu4-Gly48 segments. A set of 100 structures were generated and minimized. After that, 20 structures were randomly selected to represent all the conformational space covered by the free segments.

Binding studies of a substrate analog by NMR

The oligo d[UGGUGGUGG], abbreviated as $d(UGG)_3$, was purchased from Thermo-Fischer Scientific (Ulm, Germany). The NMR sample contained $d(UGG)_3$ and ^{15}N-ONCFLG at 0.63 and 0.32 mM concentration, respectively. Different pH values were tested: 5.19, 6.35, 7.25 and 8.97. 1D ^{1}H, 2D ^{1}H-^{15}N HSQC, 2D ^{1}H TOCSY and NOESY, and 3D ^{1}H-^{15}N HSQC-TOCSY and HSQC-NOESY spectra were recorded.

SUPPLEMENTARY RESULTS

Interactions of the d(UGG)$_3$ substrate analog with ONCFLG **variant**

To experimentally evaluate whether the differences observed for the active site residues in ONCFLG variant in comparison to the 1ONC and 2I5S structures could depend on the interaction with the substrate, we decided to test the ability of d(UGG)$_3$ a nonhydrolyzeable substrate analog to bind to ONCFLG variant.

The formation of a white precipitate was observed immediately upon mixing d(UGG)$_3$ with ^{15}N-ONCFLG at pH 5.19. Following centrifugation of the NMR tube, an HSQC spectrum was recorded whose peaks have essentially the same chemical shifts as ^{15}N-ONCFLG in the absence of d(UGG)$_3$. This suggests that d(UGG)$_3$ and some ^{15}N-ONCFLG combine to form an insoluble complex, but that excess ^{15}N-ONCFLG remains in solution. Since ONCFLG variant is positively charged at pH 5.19, but is expected to become negatively charged above pH 8, we reasoned that raising the pH could weaken favorable but putatively unspecific electrostatic interactions between d(UGG)$_3$ and ^{15}N-ONCFLG and permit the recognition and binding of the oligo to the active site. The precipitate persisted upon raising the pH to 6.35, but dissolved at pH 7.25, yielding a transparent solution.

Taking advantage of the information contained in the 3D ^{1}H-^{15}N HSQC-TOCSY and HSQC-NOESY spectra recorded at pH 7.25, and previous assignments obtained at pH 5.2 [13], we readily assigned the 2D ^{1}H-^{15}N HSQC spectra and most of the peaks in the 2D ^{1}H NOESY. The 2D ^{1}H-^{15}N HSQC spectra of ^{15}N-ONCFLG (backbone region) at pH 7.25–7.28 with or without d(UGG)$_3$ were found to be essentially the same. In addition, based on the results of the ^{1}H-^{15}N HSQC experiment designed to reveal

crosspeaks among the Nδ_1, Hε_1, Nε_2 and Hδ_2 atoms of the samples' His imidazole rings, His77 was found to be predominately in the charged state, and His58 was found to be predominately neutral in the H-Nδ_1 tautomer. The results for His25 are less conclusive, but suggest its imidazole ring is chiefly neutral and adopts a mixture of tautomeric states under these conditions. Very similar results were observed for the same experiment performed on ^{15}N-ONCFLG in the presence of d(UGG)$_3$, despite the expectation that binding of d(UGG)$_3$ should modify the chemical shifts of involved atoms and fix the tautomeric state of the active site histidines. Only the ^{1}Hα nuclei belonging to Phe54 and Gln55 near the active site were substantially affected. These results contrast with the strong perturbation of the chemical shifts of nuclei at the active site of RNase A, including the catalytic His, after substrate analog binding [23]. Finally, no intermolecular NOEs between d(UGG)$_3$ and ^{15}N-ONCFLG could be unambiguously identified, whereas over twenty had been detected between RNase A and its substrate analogs.

Interestingly, it has been found that the K_M of RNA substrates binding to ONC and the K_D of substrate analogs are relatively high (in the 0.1 mM range) and account in large measure for its relatively low ribonucleolytic activity compared to RNase A [24]. The K_D in ONCFLG variant could be even higher due to the presence of the Q1S substitution and the presence of additional residues at the termini either in the intact or cleaved linker. Since substrate binding by ONC is weakened in the presence of salt according to those authors, we lowered the ionic strength and repeated the NMR experiments at pH 7.90 and 8.97. Once again, no unambiguous intermolecular NOEs could be identified and only a few significant chemical shift differences were measured. Under these conditions, His58 is still chiefly in the H-Nδ_1 tautomer. Both His25 and

His77 are largely neutral and appear to equilibrate between the H-Nδ_1 and H-Nε_2 tautomers.

SUPPLEMENTARY REFERENCES

1. Humphrey W, Dalke A, Schulten K (1996) VMD: visual molecular dynamics. J Mol Graph 14: 33-38, 27-38.
2. Mosimann SC, Ardelt W, James MN (1994) Refined 1.7 A X-ray crystallographic structure of P-30 protein, an amphibian ribonuclease with anti-tumor activity. J Mol Biol 236: 1141-1153.
3. Guex N, Peitsch MC (1997) SWISS-MODEL and the Swiss-PdbViewer: an environment for comparative protein modeling. Electrophoresis 18: 2714-2723.
4. Leland PA, Staniszewski KE, Kim B, Raines RT (2000) A synapomorphic disulfide bond is critical for the conformational stability and cytotoxicity of an amphibian ribonuclease. FEBS Lett 477: 203-207.
5. Plainkum P, Fuchs SM, Wiyakrutta S, Raines RT (2003) Creation of a zymogen. Nat Struct Biol 10: 115-119.
6. Ribó M, Bosch M, Torrent G, Benito A, Beaumelle B, et al. (2004) Quantitative analysis, using MALDI-TOF mass spectrometry, of the N-terminal hydrolysis and cyclization reactions of the activation process of onconase. Eur J Biochem 271: 1163-1171.
7. Rodriguez M, Torrent G, Bosch M, Rayne F, Dubremetz JF, et al. (2007) Intracellular pathway of Onconase that enables its delivery to the cytosol. J Cell Sci 120: 1405-1411.
8. Bradford MM (1976) A rapid and sensitive method for the quantitation of microgram quantities of protein utilizing the principle of protein-dye binding. Anal Biochem 72: 248-254.
9. Matayoshi ED, Wang GT, Krafft GA, Erickson J (1990) Novel fluorogenic substrates for assaying retroviral proteases by resonance energy transfer. Science 247: 954-958.
10. Liu Y, Kati W, Chen CM, Tripathi R, Molla A, et al. (1999) Use of a fluorescence plate reader for measuring kinetic parameters with inner filter effect correction. Anal Biochem 267: 331-335.
11. Kelemen BR, Raines RT (1999) Extending the limits to enzymatic catalysis: diffusion of ribonuclease A in one dimension. Biochemistry 38: 5302-5307.
12. Park C, Kelemen BR, Klink TA, Sweeney RY, Behlke MA, et al. (2001) Fast, facile, hypersensitive assays for ribonucleolytic activity. Methods Enzymol 341: 81-94.
13. Serrano S, Callís M, Vilanova M, Benito A, Laurents DV, et al. (2012) ^{1}H, ^{13}C and 15N resonance assignments of the Onconase FL-G zymogen. Biomol NMR Assign in press.
14. Garcia-Mayoral MF, Castaño R, Fanarraga ML, Zabala JC, Rico M, et al. (2011) The solution structure of the N-terminal domain of human tubulin binding factor C reveals a platform for tubulin interaction. PLoS ONE 6.
15. Bai Y, Milne JS, Mayne L, Englander SW (1993) Primary structure effects on peptide group hydrogen exchange. Proteins 17: 75-86.

16. Bruix M, Ribó M, Benito A, Laurents DV, Rico M, et al. (2008) Destabilizing mutations alter the hydrogen exchange mechanism in ribonuclease A. Biophys J 94: 2297-2305.
17. Goddard TD, Kneller DG (2005) Sparky 3, University of California, San Francisco.
18. Koradi R, Billeter M, Wuthrich K (1996) MOLMOL: a program for display and analysis of macromolecular structures. J Mol Graph 14: 51-55, 29-32.
19. Güntert P (2004) Automated NMR structure calculation with CYANA. Methods Mol Biol 278: 353-378.
20. Shen Y, Delaglio F, Cornilescu G, Bax A (2009) TALOS+: a hybrid method for predicting protein backbone torsion angles from NMR chemical shifts. J Biomol NMR 44: 213-223.
21. Case DA, Darden, T. A., Cheatham, I., T. E., Simmerling, C. L., Wang, J., Duke, R. E., Luo, R., Merz, K. M., Pearlman, D. A., Crowley, M., Walker, R. C., Zhang, W., Wang, B., Hayik, S., Roitberg, A., Seabra, G., Wong, K. F., Paesani, F., Wu, X., Brozell, S., Tsui, V., Gohlke, H., Yang, L., Tan, C., Mongan, J., Hornak, V., Cui, G., Beroza, P., Mathews, D. H., Schafmeister, C., Ross, W. S. and Kollman, P. A. (2006) AMBER 9, University of California, San Francisco. .
22. Laskowski RA, Rullmannn JA, MacArthur MW, Kaptein R, Thornton JM (1996) AQUA and PROCHECK-NMR: programs for checking the quality of protein structures solved by NMR. J Biomol NMR 8: 477-486.
23. Toiron C, Gonzalez C, Bruix M, Rico M (1996) Three-dimensional structure of the complexes of ribonuclease A with 2',5'-CpA and 3',5'-d(CpA) in aqueous solution, as obtained by NMR and restrained molecular dynamics. Protein Sci 5: 1633-1647.
24. Lee JE, Raines RT (2003) Contribution of active-site residues to the function of onconase, a ribonuclease with antitumoral activity. Biochemistry 42: 11443-11450.
